# From Demyelination to Intervention: Natural Compounds as Emerging Therapeutic Targets in Multiple Sclerosis Neuroinflammation

**DOI:** 10.1155/jimr/6333512

**Published:** 2026-03-26

**Authors:** Zhiyong Long, Qianyue Yang, Yonghe Wu, Lifei Wan, Liuting Zeng, Lingyun Sun

**Affiliations:** ^1^ Department of Physical Medicine and Rehabilitation, The Affiliated Panyu Central Hospital of Guangzhou Medical University, Guangzhou, China; ^2^ Department of Rheumatology and Immunology, Nanjing Drum Tower Hospital Clinical College of Nanjing Medical University, Nanjing, China, njmu.edu.cn; ^3^ Department of Rheumatology, The First People’s Hospital of Changde City, Changde, China, yy.changde.gov.cn/; ^4^ Department of Rheumatology and Immunology, Nanjing Drum Tower Hospital, Chinese Academy of Medical Sciences and Peking Union Medical College, Nanjing, China, pumc.edu.cn

**Keywords:** demyelination, experimental autoimmune encephalomyelitis, multiple sclerosis, natural compounds, neuroinflammation, therapeutic target, translational research

## Abstract

Multiple sclerosis (MS) is a chronic immune‐mediated inflammatory demyelinating disease of the central nervous system (CNS), characterized by multifocal lesions, axonal damage, and progressive neurological dysfunction, imposing a heavy burden on patients and healthcare systems. While T lymphocytes have long been regarded as central to MS pathogenesis, accumulating evidence underscores the pivotal role of B lymphocytes and dysregulated cytokine networks (e.g., Th17/IL‐17 and NF‐κB pathways) in driving disease initiation and progression. Despite advances in symptomatic management and disease‐modifying therapies (DMTs), clinical interventions for MS remain constrained by severe side effects, drug dependency, and suboptimal long‐term efficacy, highlighting an urgent unmet need for novel therapeutic strategies. Natural compounds (e.g., alkaloids, flavonoids, terpenoids, and polyphenols) have emerged as promising candidates owing to their inherent biocompatibility, favorable safety profiles, and multi‐targeted regulatory effects on neuroinflammation. This review critically synthesizes and evaluates current evidence on the therapeutic potential of natural compounds in MS, with a focus on cross‐compound class integration of core common pathways (e.g., NF‐κB inhibition and Th17/Treg balance modulation) rather than isolated mechanism descriptions. We explicitly compare the strength of evidence across different natural compounds, clarifying which findings are well‐substantiated versus preliminary. Notably, we emphasize the inherent limitations of experimental autoimmune encephalomyelitis (EAE) models in recapitulating human MS pathophysiology (e.g., interspecies immune divergence and disease heterogeneity) and integrate or at least reference relevant clinical/epidemiological evidence to enhance translational relevance. Finally, we outline key future perspectives, including the integration of natural products with existing DMTs, challenges in clinical trial design, and prospects of combination therapies, aiming to provide a directional framework for advancing natural compounds from preclinical exploration to actionable clinical therapeutic targets in MS and enhancing the translational impact of this field.

## 1. Introduction

Multiple sclerosis (MS) is a prototypically heterogeneous chronic autoimmune neuroinflammatory disease characterized by inflammatory demyelination, glial scar formation, and progressive axonal degeneration in the central nervous system (CNS), with lesions preferentially localizing to the periventricular white matter, optic nerves, spinal cord, brainstem, and cerebellum [1, 2]. Beyond the canonical pathological features of focal demyelination and glial proliferation, accumulating evidence highlights that axonal damage—once considered a secondary consequence—occurs early in disease progression and is the primary determinant of long‐term neurological disability [3], challenging the traditional view of “relatively preserved axons” in early MS pathology. Clinically, MS presents with remarkable phenotypic diversity, with symptoms spanning visual impairment, diplopia, motor dysfunction, sensory disturbances, ataxia, and bladder/rectal dysfunction [4]; this heterogeneity is further reflected in its four major clinical subtypes: relapsing‐remitting MS (RRMS), primary progressive MS (PPMS), secondary progressive MS (SPMS), and progressive relapsing MS (PRMS), among which RRMS accounts for ~85% of initial diagnoses [5, 6]. The unpredictability of onset age, initial symptom profiles, relapse frequency, and progression rate across subtypes poses substantial challenges to early diagnosis, therapeutic stratification, and precision medicine in MS management.

The etiopathogenesis of MS remains incompletely elucidated, but is widely recognized as a complex interplay between genetic susceptibility (e.g., HLA‐DRB1 ^∗^15 haplotype) and environmental triggers (e.g., Epstein‐Barr virus infection, vitamin D deficiency, smoking) that disrupt immune homeostasis [7, 8]. A defining feature of MS is the dysregulation of both cellular and humoral immunity, which drives a multi‐cellular immune network dysregulation in both peripheral and CNS compartments. Beyond the well‐characterized crosstalk between B and T lymphocytes—where B cell‐derived autoantibodies, cytokine secretion, and antigen presentation synergize with T cell subsets (e.g., Th1, Th17) to promote neuroinflammation—macrophages, microglia, mast cells, and natural killer cells collectively contribute to the pathogenic cascade by mediating blood–brain barrier (BBB) disruption, myelin phagocytosis, and neurotoxic mediator release [9–11]. Notably, shared pro‐inflammatory signaling pathways (e.g., NF‐κB, JAK/STAT) are recurrently dysregulated across these immune cell subsets, representing potential convergent therapeutic targets; however, most existing reviews have described these cellular mechanisms in isolation, failing to capture the integrative network dynamics underlying MS progression.

Clinically, MS remains incurable, with current therapeutic strategies primarily focused on symptom alleviation rather than disease modification. Corticosteroids and disease‐modifying drugs (DMDs) can mitigate relapse severity and frequency in RRMS patients, but are plagued by substantial adverse effects (e.g., immunosuppression and metabolic disorders) and demonstrate limited efficacy in halting neurodegenerative progression in SPMS and PPMS—subtypes that account for the majority of long‐term disability [12, 13]. This therapeutic gap underscores an urgent unmet clinical need for novel, well‐tolerated agents that target the core pathogenic mechanisms of MS, particularly those driving progressive disease.

Experimental autoimmune encephalomyelitis (EAE)—the most widely used preclinical model of MS—has provided invaluable insights into the immunopathogenesis of neuroinflammation and demyelination [14, 15]. However, it is critical to acknowledge the inherent limitations of EAE in recapitulating human MS: EAE typically manifests as a monophasic or relapsing‐remitting phenotype that poorly mimics progressive MS subtypes, lacks the multifocal lesion dissemination characteristic of human MS, and exhibits interspecies differences in immune cell function and CNS immune privilege [15]. These limitations have hindered the translation of EAE‐derived findings to clinical practice, emphasizing the need for critical evaluation of preclinical evidence and integration of clinical/epidemiological insights when exploring therapeutic candidates.

Natural compounds—including *Tripterygium wilfordii*‐derived components, berberine, demethoxycurcumin, and gingerol—have emerged as promising therapeutic candidates for MS due to their extensive natural sources, low cost, favorable safety profiles, and inherent multi‐targeted regulatory effects on neuroinflammation [16, 17]. Over the past decade, a growing body of evidence has highlighted their potential to modulate key pathogenic pathways in EAE, but existing literature is fragmented: mechanisms are often described in isolation across compound classes, and the strength of evidence (e.g., well‐validated vs., preliminary findings) remains unstratified. To address these gaps, this review systematically synthesizes and critically evaluates the latest advances (2014–2024) in natural compound‐based interventions for MS, with a focus on cross‐compound class integration of shared and compound‐specific mechanisms within the context of the MS multi‐cellular immune network.

The literature search for this review was conducted across three core databases (PubMed, Web of Science, and Embase) with predefined inclusion/exclusion criteria: included studies were peer‐reviewed preclinical (EAE model) or clinical studies exploring natural compounds (alkaloids, flavonoids, terpenoids, and polyphenols) in MS‐related neuroinflammation; excluded studies were review articles, case reports, or studies focusing on synthetic derivatives of natural compounds. We explicitly compare the strength of evidence across different natural compounds, clarify preliminary findings requiring further validation, and contextualize EAE‐derived data with relevant clinical/epidemiological evidence to enhance translational relevance.

**Figure 1:**
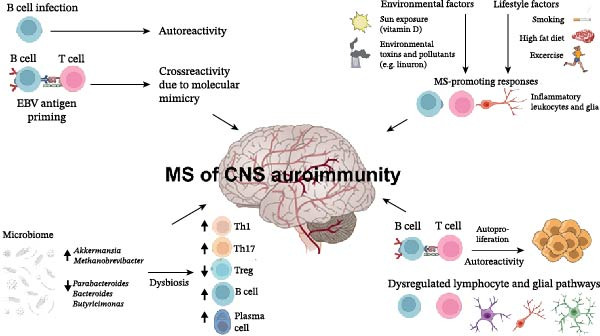
Summary of the molecular mechanisms of MS.

This review is structured to first elaborate on the integrative molecular and cellular mechanisms underlying MS neuroinflammation, emphasizing convergent pathogenic pathways across immune cell subsets. Subsequently, we discuss the regulatory effects of natural compounds on these pathways in EAE models, with a focus on integrating shared mechanisms (e.g., NF‐κB inhibition, Th17/Treg balance restoration, and microglial M1/M2 polarization regulation) to avoid redundant descriptions. Finally, we conclude with a future outlook section addressing critical challenges in translating natural compounds to clinical practice—including the development of subtype‐specific interventions, rational combination with existing DMDs, and optimizing clinical trial design—and highlight the prospects of natural compounds in advancing precision medicine for MS. By bridging preclinical mechanistic insights with clinical translational gaps, this review aims to provide a nuanced, systems‐level perspective on natural compounds as potential therapeutic modulators of MS neuroinflammation, while addressing the key limitations identified in existing literature, and thus offering actionable guidance for future preclinical and clinical research in this field (Figure 1).

## 2. Molecular Mechanisms of MS

### 2.1. Cytokines

The progression of MS involves various cytokines, including IFN‐α, IFN‐γ, IFN‐β, TGF‐β, IL‐10, IL‐12, and IL‐33. Among them, IFN‐α, IFN‐γ, IL‐12, and IL‐33 act as pro‐inflammatory mediators, while TGF‐β and IL‐10 function as inhibitory cytokines [18, 19]. TNF‐α can affect the distribution of tight junctions and adhesion molecules. The combination of TNF‐α and IFN‐γ can change the structure of adhesion proteins, leading to the aggregation of endothelial cells and reducing the permeability of the BBB, thus promoting the migration of self‐reactive T cells and B cells across the BBB [20]. Additionally, cytokines can increase the expression of adhesion molecules such as ICAM‐1, VCAM‐1, and selectins, which are necessary for leukocyte transmigration. IL‐6 can increase the expression of VCAM‐1 and promote the recruitment of leukocytes to the spinal cord. Cytokines associated with the activation of TH17 lymphocytes also contribute to the transmigration of lymphocytes across the BBB. Endothelial cells express IL‐17 receptors and IL‐22 receptors, promoting the disruption of the BBB integrity in MS patients [21, 22]. IL‐17 may promote lymphocyte migration by increasing the expression of C–C chemokine ligands (CCL) and CCL8 in endothelial cells. Elevated levels of these chemokines have been observed in the serum of MS patients [21, 22].

**Figure 2:**
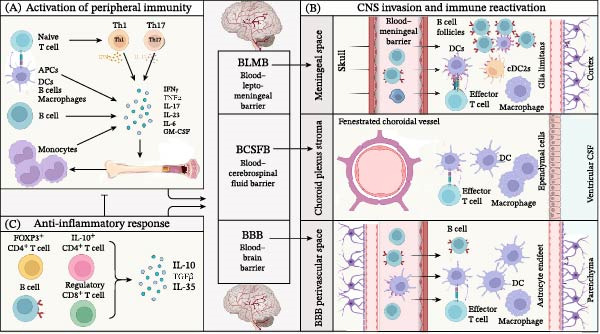
Immune mechanisms in multiple sclerosis (MS). Schematic representation of the peripheral and central immune processes driving MS pathogenesis. (A) Activation of peripheral immunity: naive T cells differentiate into pro‐inflammatory Th1 and Th17 subsets, while APCs, B cells, and monocytes secrete pro‐inflammatory cytokines (IFN‐γ, TNF‐α, IL‐17, IL‐23, and GM‐CSF), priming the systemic immune response for CNS infiltration. (B) CNS invasion and immune reactivation: effector T cells, B cells, DCs, and macrophages traverse the blood–meningeal barrier (BLMB), blood–cerebrospinal fluid barrier (BCSFB), and blood–brain barrier (BBB), reactivating within the meninges, choroid plexus, and perivascular spaces to drive local neuroinflammation. (C) Anti‐inflammatory response: regulatory T cells (FOXP3^+^ CD4^+^ and CD8^+^) and anti‐inflammatory B cells produce inhibitory cytokines (IL‐10, TGF‐β, and IL‐35) to counteract pro‐inflammatory signaling and modulate disease progression. APCs, antigen‐presenting cells; BBB, blood–brain barrier; BCSFB, blood–cerebrospinal fluid barrier; BLMB, blood–leptomeningeal barrier; DCs, dendritic cells; GM‐CSF, granulocyte‐macrophage colony‐stimulating factor; IFN, interferon; IL, interleukin; TGF‐β, transforming growth factor‐β; TNF‐α, tumor necrosis factor‐α.

Research has shown that Th17 cells are closely associated with the development of experimental autoimmune EAE, and IFN‐α can induce the production of Th17 cells. It can also activate cytotoxic T cells and NK cells, induce the expression of human leukocyte antigen class I, and trigger cellular apoptosis, resulting in the destruction of oligodendrocytes and myelin sheaths. The levels of IFN‐γ in the serum and cerebrospinal fluid (CSF) of active MS patients are significantly increased and gradually return to normal with disease remission, suggesting that IFN‐γ is a pro‐inflammatory cytokine in the pathogenesis of MS and can be used as an indicator of disease activity [23–26]. IL‐12, composed of the p35 and p40 subunits, can induce the proliferation of naive and memory T cells and the production of IFN‐γ during this process. It can also activate signaling and transcription factors, as well as stimulate the production of IL‐12 by monocytes [27]. Studies have found that the inhibition of IL‐12 secretion in the CNS can suppress the progression of EAE, indicating that IL‐12 is a pro‐inflammatory cytokine in the process of MS [28]. The role of TGF‐β in MS is less studied, but observations suggest that its activation may be associated with worsening inflammation in early‐stage relapsing‐remitting patients. In the EAE model, the administration of TGF‐β blockers can inhibit disease progression, while its administration can exacerbate the disease [29]. This suggests that TGF‐β may play a role in the chronic stage of the inflammatory response. IL‐33, a recently discovered cytokine, is expressed in various cells, including endothelial cells, epithelial cells, smooth muscle cells, macrophages, and astrocytes in the CNS. IL‐33 expression is significantly increased in spinal cord astrocytes in the EAE model. Treatment with IL‐33 antibodies can downregulate the levels of IFN‐γ and IL‐17 in the serum and inhibit the progression of EAE. Conversely, treatment with recombinant IL‐33 leads to increased IFN‐γ and IL‐17 levels, suggesting the involvement of IL‐33 in the pathogenesis of EAE [30]. Cytokines have widespread expression in the CNS and play complex roles in the disease process of MS. By intervening in the expression of certain cytokines, new approaches for clinical treatment may be explored. Moreover, the discovery of new cytokines and their biological characteristics provides new avenues for studying immune mechanisms in MS. In summary, the cytokine network exerts a dual regulatory role in MS pathogenesis by mediating BBB disruption, immune cell infiltration, and myelin damage through the coordination of pro‐inflammatory and inhibitory cytokines. However, current research has obvious limitations: most mechanistic conclusions rely heavily on EAE models, and the translational validity of these findings to human MS (especially progressive subtypes) remains insufficiently verified; the role of individual cytokines (e.g., TGF‐β) is stage‐dependent and controversial, lacking large‐sample clinical evidence to clarify their precise functional patterns; furthermore, existing studies tend to focus on single cytokines rather than exploring the synergistic or antagonistic interactions within the entire cytokine network. These gaps indicate that future research should prioritize integrating preclinical data with clinical evidence to decipher the dynamic balance of the cytokine network, which is crucial for translating cytokine‐targeted strategies into effective and subtype‐specific MS therapies. The interplay between cytokines and immune cell subsets across peripheral and CNS compartments, as depicted in Figure 2, further underscores the complexity of the MS immune landscape.

### 2.2. The Role of B Cells in the Immune Mechanisms of MS

Antibodies produced after B cell activation are immune globulins that can protect the body but also cause tissue damage by targeting self‐antigens. The role of humoral immunity in MS is not yet fully understood. It is speculated that B cells and plasma cells in the body are abnormally stimulated, leading to the production of certain antibodies against antigens that can induce MS. These cells may also damage myelin by producing self‐reactive antibodies or produce autoantibodies involved in modulating the course of MS [31]. In the EAE model, B cells act as antigen‐presenting cells (APCs) and interact with CD4+ T cells to initiate adaptive immune responses, leading to inflammatory responses against myelin antigens [32]. Inhibition of B cell immunity can result in the improvement of inflammation and clinical scores in EAE rats, indicating the significant role of B cell immunity in regulating CNS inflammation and potentially participating in the onset of MS [33]. Pathological observations have shown the presence of lymphoid follicle‐like structures in meningeal tissue of MS patients, and significant infiltration of B cells around areas of demyelination in lesions, suggesting ongoing production of mature B cells within the CNS, triggering local humoral immune responses and causing continuous damage to the cerebral cortex, which exacerbates disease progression [34]. Increased B cells have also been detected in the CSF of MS patients, and gel electrophoresis analysis has revealed the presence of oligoclonal bands (OBs) in the CSF, indicating clonal expansion of antibodies. The synthesis of intrathecal IgG in MS is one of the important features that distinguishes it from other CNS diseases. Elevated CSF protein levels have been observed in more than 70% of patients, and OBs can be detected in CSF in 95% of patients, with most OBs being of the IgG isotype, suggesting their potential role in the pathogenesis of MS [35]. Additionally, B cells can produce cytokines and antibodies that play a role in MS. B cells can differentiate into subtypes with unique cytokine profiles and produce cytokines such as TNF‐α and IFN‐γ. TNF‐α is involved in the formation of germinal centers and the generation and phenotypic switching of memory B cells [36]. Through IFN‐γ‐mediated induction, B cells can activate cytotoxic T cells, NK cells, and other immune cells, resulting in tissue damage.

Currently, regulatory B cells (Bregs) are a hot topic in the study of their role in the disease process of MS. Bregs can produce immunosuppressive cytokines such as IL‐10 and IL‐35 to induce immune tolerance and suppress inflammatory responses [37]. In EAE models, worsening of the disease and failure to achieve spontaneous remission have been observed in Breg‐deficient mice, with incomplete recovery of symptoms. This suggests that Bregs are involved in the disease remission process of EAE [38]. Bregs can inhibit the proliferation of TH1 and TH17 CD4+ T cells on one hand, and increase the expression of regulatory T cells (Tregs) and CTLA‐4 on the other hand, playing a role in regulating inflammatory responses. B cells can selectively activate Tregs through inducible co‐stimulator (ICOS) induction, and the interaction between B cells and ICOS can promote the production of IL‐10 and the formation of CD4 + CD25 + Tregs, thereby inhibiting the progression of EAE [39]. In clinical practice, the treatment of MS often involves reducing the number of B cells and their antibody production. Monoclonal antibodies targeting CD20‐expressing B cells represent an important new treatment option for MS patients. B cell depletion therapy is highly effective for relapsing forms of the disease and has been the first therapy shown to prevent disability worsening in PPMS. However, this treatment method lacks specificity in inhibiting B cell immunity and may unintentionally suppress Bregs, which potentially exacerbates the disease. Therefore, identifying the signaling pathways and specific transcription factors that regulate Bregs differentiation may provide a new therapeutic approach in clinical practice. In summary, B cells play a dual and context‐dependent role in MS pathogenesis, exerting pro‐inflammatory effects via autoantibody production, antigen presentation, and pro‐inflammatory cytokine secretion, while Bregs counteract inflammation through immunosuppressive mediators. Nevertheless, current research has notable limitations: mechanistic insights are largely derived from EAE models, whose ability to recapitulate the complexity of human MS (especially meningeal lymphoid follicle formation and progressive subtypes) is limited; the heterogeneity of B cell subsets (including Bregs) remains insufficiently defined, with unclear stage‐specific functional changes in MS; additionally, the molecular mechanisms underlying the off‐target effects of B cell depletion therapy (e.g., unintended Breg suppression) are not fully elucidated. Future research should prioritize integrating clinical pathological data with preclinical models to clarify subset‐specific B cell functions, which is critical for developing precise therapeutic strategies targeting pathogenic B cells while preserving protective Breg activity.

### 2.3. The Role of T Cells in the Immune Mechanisms of MS

Abnormal activation of immune cells is an important process in the pathogenesis of MS. Activated immune cells, cytokines, and inflammatory mediators can cross the BBB and enter the CNS, causing neuronal damage [40]. These multi‐stage immune events—encompassing peripheral T/B cell activation, CNS infiltration, and the dynamic balance between pro‐inflammatory and anti‐inflammatory responses—are comprehensively illustrated in Figure 2. Peripheral immunoreactive T and B cells are abnormally activated due to antigen stimulation, particularly the activation of myelin‐specific CD4+ T cells, which play a significant role in the pathogenesis of MS [41, 42]. CD4+ T cells are immune cells that play a crucial role in the immune response. Upon antigen stimulation, naive CD4+ T cells can be activated, proliferate, and differentiate into various types of effector Th cells that produce cytokines, including Th1 cells, Th17 cells, and Treg cells, triggering a series of immune response processes [43]. The cytokines secreted by Th1 cells can induce the differentiation of T lymphocytes into Th1 cells. The interactions among cytokines can promote the secretion of other cytokines. Trinchieri et al. suggested that IL‐12 secretion can initiate an autoimmune attack on the body, inducing EAE [44]. Th2 cells primarily secrete IL‐4, IL‐5, and transforming growth factor (TGF)‐β, which can negatively regulate immune responses and inhibit immune reactions in the CNS. Therefore, the imbalance between Th1 and Th2 cells can jointly contribute to the development of EAE [45].

#### 2.3.1. Regulation of Th1/Th2 Differentiation

Th1/Th2 are subtypes of CD4+ T cells that differentiate into helper T lymphocyte subgroups, mediating two types of immune responses, namely, Th1 and Th2, during the course of EAE. Th1 cells primarily secrete IFN‐γ, IL‐12, and TNF‐α/β, and are key immune regulatory cells in EAE [45]. The cytokines secreted by Th1 cells can promote the differentiation of T lymphocytes into Th1 cells. Moreover, cytokines can interact with each other, leading to the production of additional cytokines. It has been suggested that IL‐12 secretion can initiate an autoimmune attack on the body, inducing EAE. Th2 cells primarily secrete IL‐4, IL‐5, and TGF‐β, which negatively regulate immune responses and inhibit immune reactions in the CNS [44]. Thus, the balance between Th1 and Th2 cells plays a critical role in the development and progression of EAE. In summary, the balance of Th1/Th2 differentiation is a core regulatory node in EAE pathogenesis, with Th1‐driven pro‐inflammatory responses and Th2‐mediated anti‐inflammatory effects co‐modulating CNS neuroinflammation. However, current research has obvious limitations: most conclusions rely heavily on EAE models, and the translational relevance of Th1/Th2 balance to human MS (especially progressive subtypes) remains insufficiently validated; existing studies focus more on the phenotypic imbalance of Th1/Th2 but lack in‐depth exploration of the upstream molecular regulatory networks (e.g., key transcription factors and epigenetic modifications) that govern their differentiation; additionally, the dynamic changes of Th1/Th2 balance during different MS disease stages are not clearly defined. Future research should prioritize integrating preclinical EAE data with clinical MS specimens to clarify the stage‐specific role of Th1/Th2 balance, which is essential for developing targeted immunomodulatory strategies based on regulating Th1/Th2 differentiation.

#### 2.3.2. Regulation of Th17 Differentiation

Th17 cells are a newly discovered subset of CD4+ T cells that differentiate independently of classical helper T lymphocyte subgroups. Th17 cells primarily secrete cytokines such as IL‐17 or directly synthesize IL‐6 to induce the formation of other pro‐inflammatory factors, thereby participating in the progression of EAE [46]. Clinical studies have shown that IL‐17 mRNA expression is significantly increased in brain tissue of MS patients, and that Th17 cells can be isolated from regions of the CNS associated with focal demyelination, suggesting that IL‐17 may play a key role in the development of EAE [47]. Komiyama et al. [48] found that mice lacking IL‐17 failed to develop EAE when CD4+ T lymphocytes lacking IL‐17 were transferred into them, and that the disease model worsened when Th17 cells were directly injected compared to injection with Th1 cells [49]. Ghoreschi et al. discovered that both TGF‐β and IL‐6 can mediate Th17 differentiation, and the secretion of IL‐23 by Th1 cells plays a crucial role in the proliferation of Th17 cells. Studies have shown that IL‐23 promotes the production of IL‐17 by Th17 cells, and the use of IL‐23 p19 antibodies can lower the levels of IL‐17 and alleviate the progression of EAE [50]. Reports have also shown that TGF‐β can induce the differentiation of CD4+ T cells into Tregs, while IL‐6 and IL‐21 mediate the conversion of Tregs into Th17 cells, resulting in an imbalance between Tregs and Th17 cells and exacerbating the immune system’s inflammatory response, leading to a worsening of EAE [51]. In recent years, more and more research has indicated the significant role of Th17 cells in EAE, and with further investigation, the mechanisms of action of Th17 cells will be further revealed. In summary, Th17 cell differentiation and its crosstalk with Tregs (mediated by TGF‐β, IL‐6, etc.) are critical for driving EAE neuroinflammation, with clinical evidence of IL‐17 upregulation in MS patients supporting its pathogenic relevance. Nevertheless, current research has notable limitations: most mechanistic insights are derived from EAE models, and the specific role of Th17 cells in progressive MS subtypes (where neurodegeneration dominates) remains poorly defined; while key cytokines regulating Th17 differentiation are identified, the upstream epigenetic and transcriptional regulatory networks (e.g., RORγt co‐regulators) and their tissue‐specific modulation are insufficiently explored; additionally, the heterogeneity of Th17 subsets (e.g., pathogenic vs., non‐pathogenic Th17) in human MS is not fully characterized. Future research should focus on integrating EAE‐derived mechanisms with clinical MS samples to clarify subset‐specific Th17 functions, which is vital for developing precise therapies targeting pathogenic Th17 cells without disrupting protective immune homeostasis.

#### 2.3.3. Regulation of Th22 Differentiation

Th22 cells belong to a newly discovered subset of CD4+ T cells, and they primarily participate in the progression of EAE by secreting inflammatory cytokines such as IL‐22, IL‐13, IL‐26, and TNF‐α [52]. Research has shown that IL‐22 significantly increases in acute EAE models, suggesting that Th22 cells and IL‐22 play important roles in the progression of EAE [53]. Genetic testing in EAE models has found that both IL‐22 receptor R1 and R2 can mediate immune responses in the CNS, and their involvement in the progression of EAE can be influenced by the regulation of IL‐22 secretion [54]. Clinical studies have shown that the proportions of Th22 cells and IL‐22 are significantly elevated in the peripheral blood of relapsing MS patients [55]. The roles of Th22 cells and Tregs subsets in the mechanisms and progression of EAE are gradually becoming apparent, providing a more comprehensive theoretical basis for clinical treatment. In summary, Th22 cells are implicated in EAE progression via secreting pro‐inflammatory cytokines like IL‐22, and their elevated levels in relapsing MS patients imply potential pathogenic relevance in human MS. However, current research on Th22 differentiation regulation in MS/EAE has obvious limitations: most studies are preliminary and rely heavily on EAE models, lacking in‐depth exploration of the molecular mechanisms governing Th22 differentiation; the crosstalk between Th22 and other T cell subsets (e.g., Tregs) in MS pathogenesis remains unclear; furthermore, the role of Th22 in progressive MS subtypes and their dynamic changes across disease stages is barely investigated. Future research should prioritize validating preclinical findings in clinical MS specimens and deciphering the upstream regulatory networks of Th22 differentiation, which is essential for determining the feasibility of Th22 as a therapeutic target for MS.

#### 2.3.4. Regulation of Teff/Treg Differentiation

**Figure 3:**
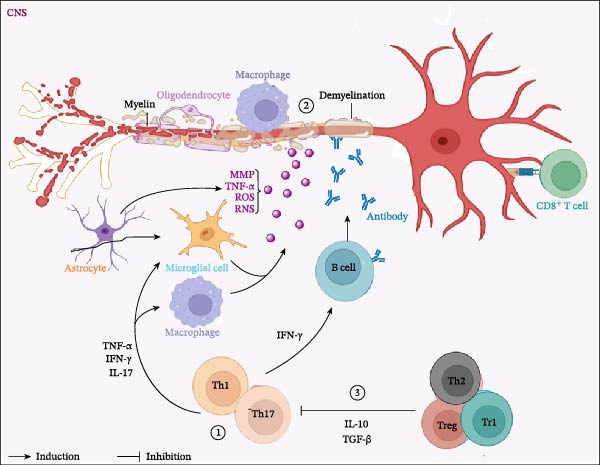
CNS damage driven by activated immune cells in the CNS.

Treg cells represent a dominant form of immune tolerance, directly suppressing the activation, proliferation, and function of effector immune cells. Various immunosuppressive mechanisms mediated by Treg cells have been revealed (Figure 1), such as the high expression of CD25 on cell surfaces, secretion of anti‐inflammatory cytokines such as IL‐10, IL‐35, and TGF‐β (in a contact‐independent manner), leading to the consumption of IL‐2 in the environment, or the trogocytosis of receptors CD80 and CD86 in APCs by CTLA‐4 (in a contact‐dependent manner) [56, 57]. Treg cells play a crucial regulatory role in peripheral immune tolerance during the progression of EAE, inhibiting excessive immune responses caused by effector T (Teff) cells and avoiding tissue damage. Villegas et al. [58] found that maintaining a dynamic balance between Teff cells and Treg cells is a key factor in maintaining peripheral immune homeostasis, and an imbalance between the two may contribute to the onset of EAE. Studies have shown that MOG‐specific Treg cells engineered with a chimeric antigen receptor (CAR) significantly reduce disease scores in an EAE‐MS mouse model [59, 60] (Figure 3). In summary, the dynamic balance of Teff/Treg differentiation is a core checkpoint for maintaining immune homeostasis in EAE, with Treg cells exerting protective effects via multiple contact‐dependent and independent immunosuppressive mechanisms, and CAR‐Treg strategies showing promising preclinical potential. Nevertheless, current research has notable limitations: most mechanistic insights and therapeutic explorations are based on EAE models, and the translational relevance of Teff/Treg balance to different MS subtypes (especially progressive forms) remains to be validated; the heterogeneity of Treg subsets (e.g., natural vs., induced Tregs) and their functional stability in MS microenvironments are insufficiently characterized; additionally, the long‐term safety and efficacy of CAR‐Treg therapy in clinical MS settings (e.g., off‐target effects, persistence) are yet to be addressed. Future research should focus on integrating preclinical data with clinical MS specimens to clarify subtype‐specific Teff/Treg dynamics and optimize Treg‐based therapeutic strategies, which is vital for advancing precision immunomodulation in MS.

### 2.4. Epigenetics of MS

Epigenetics is a branch of genetics that studies changes in gene expression without altering the DNA sequence, including modifications such as DNA methylation, histone post‐translational modifications, microRNAs (miRNAs), and long non‐coding RNAs (lncRNAs) [61]. The concordance rate of MS in monozygotic twins is ~25%, suggesting that epigenetics may partially contribute to the occurrence and development of MS [62].

#### 2.4.1. miRNA

miRNAs are short, conserved, single‐stranded non‐coding RNA segments about 22 nucleotides in length. They are processed into hairpin structures by Dicer and bind to the 3^′^ untranslated regions (3^′^‐UTRs) of target genes, leading to mRNA degradation or inhibition of translation [63]. Studies have shown differential expression of various miRNAs in MS patients. For example, miR‐155, miR‐326, miR‐125a–3p, and miR‐146a are upregulated, while miR‐18a‐5p, miR‐34a, miR‐485, miR‐708, miR‐30c, and miR‐23a are downregulated [64–72]. Our previous studies have demonstrated that miR‐125a, miR‐146b, miR‐200c, and miR‐BHRF1‐2‐5p are upregulated, while miR‐328, miR‐199a, and miR‐152 are downregulated in Chinese MS patients [73–75]. miR‐146a in MS patients selectively inhibits the inflammation‐dependent Th1 response mediated by IFN‐γ and Tregs [67, 76]. miR‐199a and miR‐18a target IL‐17A and inhibit Th17 differentiation [77]. let‐7 inhibits differentiation dependent on IL‐1R1/IL‐23R, prevents the migration of Th17 cells mediated by chemokine (C–C motif) receptor 2 (CCR2)/CCR5, and confers protection against experimental autoimmune EAE [75, 78]. Knocking out miR‐223–3p increases the number of Treg cells and reduces the activation of dendritic cells (DCs) [79, 80]. These experimental results suggest that miRNAs regulate T lymphocyte differentiation, influencing the onset, disease severity, and CNS inflammation in EAE. In summary, miRNAs serve as key epigenetic regulators in MS/EAE by modulating T lymphocyte differentiation and CNS inflammation, with consistent differential expression profiles (including in Chinese MS cohorts) supporting their pathogenic and clinical relevance. However, current research has notable limitations: most mechanistic insights rely on EAE models or in vitro experiments, and the in vivo functional specificity of individual miRNAs in human MS (especially progressive subtypes) remains unvalidated; existing studies focus predominantly on single miRNA‐target interactions, lacking systematic exploration of miRNA regulatory networks and their crosstalk with other epigenetic modifications (e.g., DNA methylation); additionally, the translational potential of miRNAs as diagnostic biomarkers or therapeutic targets is hindered by insufficient large‐sample clinical validation and unclear in vivo delivery strategies. Future research should prioritize integrating multi‐omics data from clinical MS specimens with preclinical models to decipher miRNA regulatory networks, which is essential for advancing miRNA‐based precision diagnosis and targeted therapies for MS.

#### 2.4.2. LncRNA

LncRNAs are a group of non‐coding transcripts with a length exceeding 200 nucleotides that play a crucial role in regulating gene expression [81, 82]. Recent research has shown an association between LncRNAs and MS, indicating their potential as biomarkers for predicting disease activity, progression, and prognosis [83, 84]. Studies have shown that serum levels of LincR‐EPAS1‐30As decrease in MS patients and are negatively correlated with disease severity. LincR‐EPAS1‐30As is located in the Th2‐enriched immune regulatory protein gene locus, and its significant downregulation is thought to play a role in the pathogenesis of MS [85]. In addition, serum levels of growth arrest‐specific transcript 5 (GAS5), nuclear paraspeckle assembly transcript 1 (NEAT1), taurine‐upregulated gene 1 (TUG1), and 7SK small nuclear RNA (RN7SK) are upregulated in MS patients, while plasmacytoma variant translocation 1 (PVT1), antisense transcript of Fas (FAS‐AS1), and myocardial infarction‐associated transcript (MIAT, also known as GOMAFU) are downregulated [74, 86]. Gas5 inhibits the transcription of interferon regulatory factor 4 (IRF4), thereby suppressing M2 polarization of microglia, regulating lymphocyte apoptosis and cell cycle, and participating in demyelination processes [87–89]. GOMAFU inhibits splicing and the formation of splicing complexes by binding to splicing factor 1 (SF1), contributing to the pathogenesis of EAE [90]. LncRNA HOX transcript antisense intergenic RNA (HOTAIR) is involved in the pathogenesis of EAE, and inflammation and vitamin D can regulate HOTAIR [91]. In summary, LncRNAs are emerging as important epigenetic regulators in MS/EAE pathogenesis, with distinct differential expression profiles in MS patients and diverse regulatory roles (e.g., modulating microglia polarization, lymphocyte function, and splicing processes) supporting their pathogenic and biomarker potential. Nevertheless, current research has obvious limitations: most mechanistic studies rely on EAE models or in vitro experiments, and the in vivo functional relevance of individual LncRNAs in human MS (especially progressive subtypes) remains to be validated; existing studies focus primarily on single LncRNA functions, lacking systematic exploration of LncRNA regulatory networks and their crosstalk with other epigenetic modifications (e.g., miRNAs and DNA methylation); additionally, the clinical translational value of LncRNAs is hindered by insufficient large‐sample validation, unclear stage‐specific expression patterns, and undefined molecular mechanisms underlying their regulatory effects. Future research should prioritize integrating clinical MS specimens with multi‐omics approaches to decipher LncRNA regulatory networks, which is vital for advancing the understanding of MS epigenetic mechanisms and developing LncRNA‐based diagnostic and therapeutic strategies.

#### 2.4.3. DNA Methylation

DNA methylation is an important epigenetic modification that involves adding a methyl group to the C5 position of cytosine by DNA methyltransferases (DNMTs). Ten‐eleven translocation (Tet) protein family, including TET1, TET2, and TET3, participate in the demethylation process. DNA methylation influences chromatin remodeling, regulates immune‐related gene promoter regions, and plays a crucial role in cell proliferation and differentiation. Research has shown increased hippocampal DNA methylation in MS patients, with upregulated DNMTs and downregulated TET proteins, leading to increased demyelination in the hippocampal region [92]. DNMT is negatively correlated with forkhead transcription factor O class 3a (FOXO3a) transcription levels, and the variation of the FOXO3a gene rs2253310 is associated with increased methylation and reduced neurodegeneration in relapsing MS patients [93]. Many environmental factors can cause DNA methylation changes, such as high salt intake, smoking, vitamin D deficiency, Epstein‐Barr virus infection, and obesity [94–99]. High salt intake leads to the overexpression of TET2, increased demethylation and hydroxymethylation levels of CD4+ T cells, and disruption of the BBB [98]. Smoking results in the demethylation of the aryl hydrocarbon receptor (AhR) repressor (AHRR) and inhibition of the AhR signaling pathway, exacerbating neuroinflammation [95, 100]. Reduced vitamin D levels lead to excessive methylation of genes DKK1 and Wnt5a, involved in the Wnt signaling pathway, increasing susceptibility to MS [97, 99, 101]. Obese MS patients exhibit high methylation of anti‐proliferative genes, resulting in higher monocyte counts than normal‐weight patients and accelerating disease progression [102]. Environmental factors interact with DNA methylation, impacting not only immune cells like CD4+ T cells but also regulating inflammatory signaling pathways, thereby accelerating the progression of EAE and MS. In summary, DNA methylation, as a core epigenetic modification, regulates MS/EAE pathogenesis by modulating immune‐related gene expression and interacting with environmental factors (e.g., high salt, smoking, and vitamin D deficiency) to affect immune cell function and inflammatory signaling. However, current research has notable limitations: most mechanistic insights rely on EAE models or in vitro studies, and the in vivo relevance of DNA methylation patterns (e.g., DNMT/TET balance) in human MS subtypes (especially progressive forms) remains insufficiently validated; existing studies focus more on individual gene methylation or single environmental factor interactions, lacking systematic exploration of DNA methylation regulatory networks and their crosstalk with other epigenetic modifications (e.g., miRNAs and LncRNAs); additionally, the clinical translational value is hindered by unclear stage‐specific methylation signatures and insufficient large‐sample validation of methylation‐based biomarkers or therapeutic targets. Future research should prioritize integrating clinical MS specimens with multi‐omics approaches to decipher DNA methylation regulatory networks and their environmental interactions, which is essential for deepening the understanding of MS epigenetic mechanisms and developing precision diagnostic and therapeutic strategies.

#### 2.4.4. RNA Methylation

Chemical modifications of RNA can affect gene expression, with over 60% of RNA modifications being methylation [103]. N6‐methyladenosine (m6A) is the most common mRNA methylation modification. The methyltransferase‐like (METTL) proteins catalyze m6A RNA methylation, while fat mass and obesity‐associated protein (FTO) and alk B homolog 5 (ALKBH5) can demethylate m6A‐modified bases [104, 105]. Oligodendrocytes are responsible for myelin sheath formation and play a crucial role in remyelination [106]. Studies have shown that METTL14 downregulates the splicing of neurofascin 155 (NFasc155) and regulates the stability of oligodendrocyte lineage transcription factor 2 (Olig2), affecting the differentiation and maturation of oligodendrocytes. m6A RNA modification is indispensable for oligodendrocyte development and CNS myelination [107]. However, the relationship between m6A RNA methylation and MS is still unclear and requires further research. In summary, RNA methylation (especially m6A modification) plays an indispensable role in oligodendrocyte development and CNS myelination, a process closely related to MS pathogenesis (e.g., demyelination and remyelination). Nevertheless, current research on RNA methylation in MS is in its infancy with obvious limitations: the association between RNA methylation and MS remains poorly defined, with a lack of direct clinical evidence from MS patients and mechanistic studies in EAE models; existing insights are mostly focused on m6A modification in oligodendrocyte development, lacking exploration of its regulatory effects on immune cells (a core component of MS pathogenesis) and crosstalk with other epigenetic modifications (e.g., miRNAs, DNA methylation); additionally, the specific molecular mechanisms linking RNA methylation to MS‐related demyelination or remyelination are yet to be elucidated. Future research should prioritize validating the role of RNA methylation in MS patients and EAE models, exploring its multi‐dimensional regulatory networks in MS pathogenesis, which is vital for enriching the epigenetic mechanism landscape of MS and identifying potential new therapeutic targets.

#### 2.4.5. Histone Modifications

Histones are responsible for DNA packaging and arrangement within nucleosome structures. Each histone can contain over 100 modification sites, including acetylation, methylation, phosphorylation, ubiquitination, deamination, and ADP ribosylation. Histone acetylation research is the most extensive, with histone acetyltransferases (HATs) and histone deacetylases (HDACs) being key enzymes in histone acetylation. Histone acetylation is closely associated with the regulation of oligodendrocyte development and differentiation [108]. Studies have observed decreased levels of the HDAC class III member sirtuin‐1 (SIRT1) in MS, which inhibits myelin formation. In animal experiments, SIRT1 overexpression alleviates clinical symptoms, reduces inflammatory cell infiltration, demyelination, and axonal damage in EAE [109]. Vitamin D3 recruits HDAC2 to the IL‐17A promoter region, thereby inhibiting IL‐17 transcription and delaying the onset of MS [110]. In conclusion, recent research suggests that MS development is regulated by multiple factors, including genetics and epigenetics. Epigenetic modifications and interactions play a role in remyelination and CNS inflammation in MS. In summary, histone modifications (particularly acetylation) are involved in MS/EAE pathogenesis by regulating oligodendrocyte development, myelin formation, and inflammatory responses (e.g., SIRT1‐mediated myelin protection and HDAC2‐dependent IL‐17 inhibition). However, current research has notable limitations: studies are predominantly focused on histone acetylation, with other modification types (e.g., methylation and phosphorylation) rarely explored in the context of MS; most mechanistic evidence relies on EAE models and in vitro studies, with insufficient validation of histone modification patterns and functions in human MS (especially progressive subtypes); additionally, the crosstalk between histone modifications and other epigenetic regulatory mechanisms (e.g., miRNAs and DNA methylation) in MS remains unclear, and the stage‐specific roles of histone‐modifying enzymes are not well defined. Future research should expand the scope of histone modification types studied, integrate clinical MS specimens to validate preclinical findings, and explore the synergistic regulatory networks of histone modifications with other epigenetic factors, which is essential for comprehensively understanding MS epigenetic mechanisms and developing targeted therapeutic strategies.

## 3. Treatment Drugs for MS

**Figure 4:**
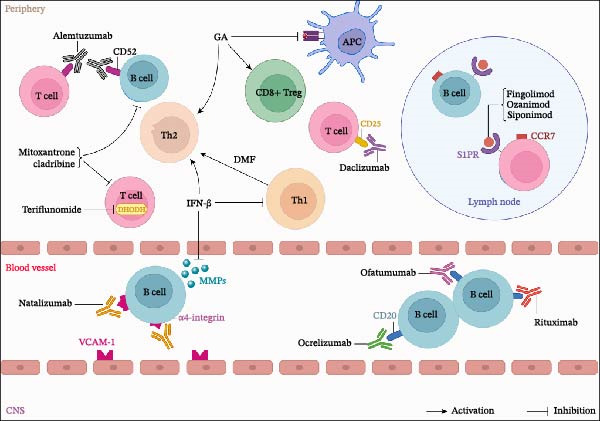
Treatment drugs for multiple sclerosis.

The treatment of MS is mainly divided into acute phase treatment and remission phase treatment [111]. Before the 1990s, the treatment of MS was limited to short courses of high‐dose glucocorticoids in the acute phase. Over the past 20 years, interferon‐beta (IFN‐β) injections and glatiramer acetate (GA) have become first‐line treatments for remission in patients with RRMS. Since 2010, with the introduction of oral disease‐modifying therapies (DMTs) and monoclonal antibodies, the treatment goal of MS has expanded from solely relieving acute symptoms to controlling disease progression [111] (Figure 4).

### 3.1. IFN‐β

IFN‐β was the first DMT approved by the United States Food and Drug Administration (FDA). It improves clinical symptoms, reduces relapse rates, and delays disease progression by reducing pro‐inflammatory cytokines and inducing the production of anti‐inflammatory cytokines that inhibit T‐cell activity [111]. IFN‐β may cause adverse reactions such as lymphocyte and platelet reduction and elevated liver enzyme levels [112]. Therefore, regular monitoring of complete blood cell count and liver function is recommended before and during treatment [113].

### 3.2. GA

GA is a mixture of polypeptides and is FDA‐approved for the treatment of RRMS. It induces the production of anti‐inflammatory B cells, enhances B‐cell biological activity, and promotes the conversion of pro‐inflammatory Th1 cells into anti‐inflammatory Th2 cells. It modulates the immune network and influences the pathogenesis of MS [114]. Additionally, GA exerts neuroprotective and neurorepair effects by promoting the release of brain‐derived neurotrophic factor (BDNF) [115]. GA has demonstrated clear efficacy in adult MS patients, reducing relapse and disability rates [116]. Adverse reactions of GA may include flushing, chest tightness, and anxiety, which are typically self‐limiting and do not require discontinuation of the medication [117].

### 3.3. Intravenous Immunoglobulin (IVIG)

IVIG is commonly used for the acute phase treatment of MS, improving clinical symptoms, reducing relapse rates, and delaying disability progression in RRMS patients [118]. Additionally, it can reduce lesion volume and the number of lesions in the brain [119]. In the treatment of MS acute attacks, the effects of IVIG are comparable to high‐dose methylprednisolone pulse therapy, but with a faster onset of action and milder adverse reactions. Symptoms such as headache, palpitations, and chills may occur within 1 h of administration, but they generally subside without specific treatment [120]. IVIG is relatively expensive and, if economically feasible, can be a preferred choice for acute phase treatment of MS.

### 3.4. Mitoxantrone

Mitoxantrone is a synthetic potent immunosuppressant that can cross the BBB and inhibit immune cell proliferation, reducing immune responses. Treatment with mitoxantrone for 3 or 6 months, followed by first‐line therapy, can improve disease assessment scores in highly active RRMS patients over a 10‐year period [121]. Common adverse reactions include nausea, menstrual irregularities, urinary tract infections, and diarrhea, with severe cases potentially leading to cardiac toxicity. Therefore, patients with a baseline left ventricular ejection fraction below the lower limit of normal should not use this medication.

### 3.5. Fingolimod

Fingolimod is a sphingosine 1‐phosphate receptor (S1PR) modulator. By binding to S1PRs on lymphocytes, it prevents their release from lymphoid tissues, thereby inhibiting immune responses [122]. In a real‐world study of RRMS patients, after 1 year of treatment with fingolimod, 83.5% of MS patients had no relapse, and 91.3% had no disability progression, demonstrating its effectiveness as a treatment for RRMS [123]. A prospective study also showed that fingolimod has lower relapse rates compared to patients switching from other first‐line treatments [124]. Common adverse reactions of fingolimod include hypertension, macular edema, and bradycardia. It is contraindicated in patients with concomitant cardiovascular and cerebrovascular diseases [125].

### 3.6. Siponimod

Siponimod is a selective S1PR modulator that binds to S1P receptor subtypes 1 and 5 on lymphocytes, leading to their internalization and degradation, ultimately preventing lymphocyte egress from lymphoid organs [126]. Siponimod is the first medication that can delay disability progression in patients with SPMS, reducing the risk of disability progression represented by whole‐brain and gray matter atrophy in SPMS patients. It also improves cognitive function in patients [127]. The main adverse reactions of siponimod include nasopharyngitis, headache, lymphocyte reduction, upper respiratory tract infections, increased alanine aminotransferase levels, pharyngitis, and insomnia. Due to its high selectivity, siponimod has minimal cardiac toxicity [128].

### 3.7. Teriflunomide

Teriflunomide is the active metabolite of the anti‐rheumatic drug leflunomide. It inhibits dihydroorotate dehydrogenase in the de novo pyrimidine synthesis pathway, reducing the proliferation of activated T cells and B cells, as well as decreasing CNS lymphocyte infiltration and demyelination, resulting in improved neurological function [129]. Teriflunomide effectively reduces the risk of relapse in patients with MS who have clinical isolated syndrome (CIS) and improves cognitive impairment in patients with relapsing MS [130]. Real‐world studies have shown that teriflunomide has good sensitivity and tolerability in RRMS patients, especially in female patients, those with low relapse rates, and those who are not severely disabled [131]. Common adverse reactions include headache, elevated alanine aminotransferase levels, diarrhea, hair loss, and nausea [132].

### 3.8. Dimethyl Fumarate (DMF)

DMF improves the inflammatory state of MS patients by decreasing the number of memory T cells and reducing the production of pro‐inflammatory Th1/Th17 cells. Its metabolites activate the nuclear transcription factor Nrf2‐dependent cytoprotective pathway, protecting neurons and exerting a therapeutic effect [133]. Studies have shown that DMF reduces lesion volume in RRMS patients and has comparable effects on reducing relapse rates and disability progression as fingolimod [134]. Adverse reactions of DMF may include nausea, diarrhea, and elevated liver enzymes. If symptoms such as difficulty breathing, swelling of the tongue or throat, characteristic of an allergic reaction and angioedema, occur, the medication should be discontinued, and immediate medical attention is required.

### 3.9. Monoclonal Antibodies

#### 3.9.1. Natalizumab

Natalizumab is a humanized monoclonal antibody against α‐4 integrin that acts as a selective adhesion molecule inhibitor. By inhibiting α‐4 integrin, it prevents immune cells from crossing the blood vessel wall, thereby preventing their arrival at affected organs [135]. In a phase III clinical trial, although natalizumab did not significantly improve EDSS scores in SPMS patients with expanding disability, it improved upper limb mobility [136]. Another study demonstrated that natalizumab reduces relapse rates and disability progression in RRMS patients, confirming its effectiveness and long‐term safety [137]. Adverse reactions include hypersensitivity and liver damage. If fever, chills, or other signs are observed, patients should be instructed to discontinue the medication [138].

#### 3.9.2. Alemtuzumab

Alemtuzumab is a humanized monoclonal antibody that targets CD52 on the surface of monocytes and lymphocytes. It inhibits the maturation of T cells, B cells, and monocytes expressing CD52, thereby reducing the number of lymphocytes and attenuating or shutting down the autoimmune attack on myelin fibers [139]. For RRMS patients with no treatment history, alemtuzumab can reduce disease relapse and exhibits better safety and efficacy compared to IFN‐β [140]. Recent real‐world studies have shown that alemtuzumab can also improve cognitive function, reduce fatigue symptoms, and lower disability rates in RRMS patients [141]. Common adverse reactions of alemtuzumab include injection site reactions, thyroid dysfunction, and upper respiratory infections.

#### 3.9.3. Daclizumab

Daclizumab is a humanized monoclonal antibody that inhibits the activation of Teff cells and regulates the survival of T cells by blocking the interaction between CD25 and IL‐2 receptor on T cells at the DAC and HYP sites, ultimately inducing T cell apoptosis [142]. RRMS patients treated with daclizumab have lower annual relapse rates and lesion burden compared to those treated with IFN‐β, but they have an increased risk of infections, rash, and liver damage [143]. Combination therapy with daclizumab and IFN‐β can reduce the number of lesions compared to IFN‐β alone [144]. Common adverse reactions of daclizumab include liver damage, immune disorders, infections, depression, and hypersensitivity reactions, making it contraindicated in patients with severe liver damage [138].

#### 3.9.4. Ocrelizumab

Ocrelizumab is a second‐generation anti‐CD20 IgG1 monoclonal antibody that can induce B cell depletion through multiple pathways while preserving CD20‐negative plasma cells [145]. Compared to IFN‐β, ocrelizumab slows down disease progression in patients with relapsing MS in the short term [146]. In the long term, early and continuous treatment with ocrelizumab benefits patients with PPMS over a longer period [147]. Ocrelizumab increases the risk of malignancies, and other adverse reactions include injection reactions and infections.

#### 3.9.5. Ofatumumab

Ofatumumab is a fully human anti‐CD20 monoclonal antibody that selectively depletes B cells by binding to small loop epitopes on CD20, without affecting other normal tissues. Intravenous administration of ofatumumab reduces relapse rates and limits disability progression in RRMS [148]. Clinical studies have shown that ofatumumab reduces relapse rates and decreases the number of lesions. Compared to teriflunomide, ofatumumab is more effective in reducing disability progression unrelated to relapse activity and annual relapse rates [149]. Adverse reactions of ofatumumab include infections, infusion reactions, and reduced immunoglobulin levels, but overall tolerability is manageable. Currently, clinical treatments for MS mainly revolve around regulating peripheral immune responses, which can only delay disease progression and cannot promote remyelination and repair of the myelin sheath. Therefore, they cannot cure MS. The pathogenesis of MS involves multiple steps. During the development of MS, T cells are excessively activated, and myelin sheaths and oligodendrocytes become targets of T cell attacks. T cells cross the BBB, leading to the release of inflammatory factors and demyelination, ultimately resulting in neurological dysfunction [150]. Clinical studies have also confirmed that neuroinflammation is a necessary condition for the formation of new lesions and causes axonal degeneration [151]. Therefore, regulating immune responses, slowing down neuroinflammation, and promoting remyelination have become the main targets and research directions in MS drug therapy in recent years. In particular, many natural compounds have shown good effects in the treatment and research of MS, suggesting that drugs targeting multiple targets in complex diseases have great application value and development prospects. In summary, MS therapeutic options have advanced substantially from acute symptom relief to chronic progression control, with oral DMTs and monoclonal antibodies improving efficacy and convenience for relapsing‐remitting subtypes. However, critical limitations persist: first, therapeutic efficacy is highly subtype‐dependent, with most agents showing limited benefits for progressive MS (e.g., PPMS), where neurodegeneration dominates; second, mechanisms are overly focused on peripheral immune suppression, lacking targeted effects on CNS remyelination and neuroprotection, rendering a cure unattainable; third, safety and accessibility issues remain prominent, including severe adverse reactions (e.g., cardiac toxicity and infection risk) of some drugs and high economic burden of agents like IVIG; finally, personalized treatment strategies are lacking, with insufficient biomarkers to guide drug selection for individual patients. Future research should prioritize developing multi‐target agents that integrate immune regulation with remyelination promotion, exploring subtype‐specific therapeutic targets, and establishing precision medicine frameworks based on clinical and molecular biomarkers, while optimizing drug safety profiles and accessibility to address unmet clinical needs in MS treatment.

## 4. Molecular Mechanisms of Natural Compounds in the Intervention of Experimental Autoimmune EAE

### 4.1. Molecular Mechanisms of Alkaloids in the Intervention of Experimental Autoimmune EAE

Matrine, a tetracyclic quinazoline alkaloid, is found in the seeds and roots of *Sophora flavescens* Alt. Matrine at a dose of 150–200 mg/kg significantly inhibits the secretion of inflammatory cytokines by Th1/Th17 cells in EAE mice, upregulates the expression of neurotrophic factor NT3, and thereby prevents inflammation and demyelination in EAE mice. It also inhibits the necrosis of CNS oligodendrocytes and improves the neurological function score in EAE mice [152, 153]. Matrine regulates the polarization of microglia from M1 to M2 phenotype and repairs p75 neurotrophic factor receptor (p75NTR) and neurotrophic factor precursor (proNGF) by inhibiting the release of interleukin‐33 (IL‐33) and soluble ST2 (sST2) in the brain tissue of EAE mice, exerting its therapeutic effects [154, 155]. Matrine reduces demyelination in EAE rats, inhibits extravasation of Evans blue (EB) through the BBB, dose‐dependently increases the mRNA expression of collagen IV and tight junction protein‐1 (ZO‐1), and downregulates matrix metalloproteinase‐9 (MMP‐9)/MMP‐2 while upregulating tissue inhibitors of metalloproteinase‐1 (TIMP‐1)/TIMP‐2. Matrine at a dose of 250 mg/kg induced a significantly greater increase in TIMP‐1 expression compared to dexamethasone, resulting in a significant improvement in clinical symptoms in EAE rats [156]. A recent seminal study [157] demonstrated that combined oxymatrine treatment significantly reduced neurological deficit scores, ameliorated immune cell infiltration and demyelination, and concomitantly rectified stroke‐induced gut dysbiosis in MS. Specifically, this intervention notably diminished the species richness and abundance of intestinal pathogenic and opportunistic pathogens, while increasing those of probiotic genera (e.g., *Bifidobacterium* and *Lactobacillus*). It also modulated the concentrations of various short‐chain fatty acids (SCFAs), suppressed inflammatory responses in both intestinal and brain tissues, markedly upregulated the mRNA expression of tight junction proteins (ZO‐1 and Occludin) in the gut–brain axis, and substantially enhanced intestinal barrier and BBB integrity—ultimately exerting a therapeutic effect on MS via the gut–brain axis regulatory pathway. Furthermore, fecal microbiota transplantation (FMT) from oxymatrine‐treated experimental autoimmune EAE mice alleviated clinical severity and weight loss in recipient mice, mitigated inflammatory infiltration and demyelination in spinal cord tissues, reduced the frequencies of Th2 and Th17 cells in the spleen and lymph nodes, increased Treg cell populations, decreased peripheral blood IL‐17A levels, and elevated IL‐10 concentrations. These findings further corroborate oxymatrine’s pivotal role in regulating the intestinal microenvironment of MS, providing critical preclinical evidence for its potential as a gut‐targeted therapeutic agent in MS management [157].

Berberine, an isoquinoline alkaloid, is widely found in plants of the Berberidaceae and Rutaceae families, with anti‐inflammatory and neuroprotective properties. Berberine has been found to reduce clinical and pathological parameters in EAE mice, significantly downregulate and inhibit the expression and activation of MMP‐9 in the CSF of EAE mice, but has no significant effect on MMP‐2. It also reduces the permeability of the BBB, inhibits lymphocyte infiltration into the CNS, and alleviates inflammation [158]. Jiang et al. [159] reported that berberine protects neurons from damage by inhibiting the activity of MMP‐9 and degradation of laminin. These findings suggest that berberine may be a potential therapeutic agent for MS.

Sinomenine, isolated from plants of the Illigera Bl. genus, is a biologically active alkaloid with a chemical structure similar to morphine and is often used as an immunosuppressive drug for the treatment of rheumatoid arthritis. Zeng et al. [160] found that sinomenine reduces cellular infiltration in the spinal cord of EAE‐induced Lewis rats in a dose‐dependent manner and inhibits the secretion and expression of inflammatory cytokines tumor necrosis factor‐α (TNF‐α) and interferon‐γ (IFN‐γ), as well as significantly inhibiting the mRNA expression levels of chemokines CCL2, RANTES, macrophage inflammatory protein‐1α (MIP‐1α), and monocyte chemotactic protein‐1 (MCP‐1). Gu et al. [161] found that sinomenine at a dose of 50–200 mg/kg exerts its effects by inhibiting inducible nitric oxide synthase (iNOS), TNF‐γ, and transcription factors T‐bet mRNA and protein expression in the spinal cord tissue of EAE rats. In vitro experiments showed that sinomenine has no direct inhibitory effect on iNOS mRNA and protein expression in astrocytes, suggesting that sinomenine exerts its therapeutic effects by reducing iNOS mRNA levels through the inhibition of the IFN‐γ and T‐bet pathways [160, 161].

Piplartine, also known as piperlongumine, is an amide alkaloid extracted from plants of the *Piper sylvaticum* and *P. tuberculatum* species in the Piperaceae family and exhibits various pharmacological properties, such as anti‐tumor, anti‐inflammatory, and analgesic effects. Kim et al. [162] found that piplartine at a concentration of 5 μmol/L inhibits the inflammatory response in LPS‐induced BV2 microglial cells, reducing the expression of iNOS, cyclooxygenase‐2 (COX‐2), TNF‐α, and IL‐6. Gu et al. [163] found that piplartine at a dose of 1.5–3 mg/kg alleviates paralysis and pathological changes in neural tissues of EAE mice, inhibits pathway activation in demyelination and astrocyte/microglia, reduces immune cell infiltration, and downregulates the levels of inflammatory cytokines iNOS, COX‐2, TNF‐α, and IFN‐γ. The underlying mechanism suggests that piplartine acts by inhibiting the translocation of nuclear transcription factor‐κB (NF‐κB) family transcription factors p50 and p65 to the nucleus, as well as IκB kinase phosphorylation [162, 163].

Huperzine A, primarily found in *Huperzia serrata*, a member of the Huperziaceae family, is a second‐generation reversible acetylcholinesterase inhibitor that has been shown to have significant therapeutic effects in Alzheimer’s disease (AD). Wang et al. [164] demonstrated that Huperzine A reduces the T cell‐mediated neuroinflammatory response, lowering the levels of inflammatory cytokines IFN‐γ, IL‐17, chemokines MCP‐1, RANTES, and TNF‐like weak inducer of apoptosis (TWEAK) in the brain tissue of EAE mice, and improves the severity of brain tissue lesions. Tian et al. [165] found that Huperzine A at a dose of 0.1 mg/(kg·d) significantly alleviates inflammation, demyelination, and axonal damage in the spinal cord of EAE mice, with its therapeutic effects attributed to the inhibition of MMP‐9, TNF‐γ, transcription factor T‐bet mRNA, and protein expression in the spinal cord tissue. The mechanism of action involves the inhibition of CCL2, IL‐6, TNF‐α, and IL‐1β secretion within the tissue [164, 165].

Anatabine, an alkaloid isolated from plants of the Solanaceae family, including Nicotiana species, exhibits anti‐inflammatory and neurostimulatory effects. Paris et al. [166] and Paris et al. [167] found that anatabine inhibits the transcriptional signaling of nuclear transcription factors and activation of transcription activation factor 3 (STAT3) and NF‐κB in LPS‐induced microglial cells and monocytes in vitro, reducing the levels of phosphorylated NF‐κB and STAT3. Furthermore, anatabine crosses the BBB, reduces Th1 and Th17 levels, inhibits NF‐κB family transcription factor p65, and inhibits STAT3 phosphorylation. Anatabine at a dose of 20 mg/(kg·d) significantly improves neurological deficits in EAE mice [166, 167]. Betaine, a natural methyl donor compound, exhibits potent neuroprotective properties. As reported by Rahdar et al. [168], compared with the MS group, betaine treatment effectively prevented and reversed adverse behavioral phenotypes. Specifically, betaine administration afforded protection against cerebellar demyelination, neuronal degeneration, and Purkinje cell loss, thereby mitigating cuprizone (CPZ)‐induced demyelination. Mechanistically, betaine downregulated the protein levels of ESR‐related proteins in the cerebellum of MS rats while concurrently elevating the expression of antioxidant‐related enzymes in this brain region. Collectively, these findings indicate that oral betaine may serve as a novel adjuvant therapeutic strategy targeting cerebellar dysfunction in animal models of MS.

**Figure 5:**
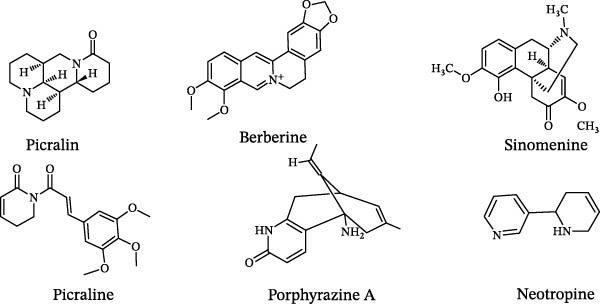
Chemical structures of alkaloids with EAE activity.

The chemical structures of alkaloids with EAE activity are shown in Figure 5.

### 4.2. Molecular Mechanisms of Flavonoids in the Intervention of Experimental Autoimmune EAE

Baicalein, a polyhydroxy flavone extracted from the root of Scutellaria baicalensis Georgi, a member of the Lamiaceae family, exhibits biological activities such as anticancer, antiviral, and antitumor effects. Zhang et al. [169] found that intraperitoneal administration of baicalein at a dose of 100 mg/(kg·d) has therapeutic and preventive effects on EAE mice. Baicalein can reduce CNS inflammatory cell infiltration, inhibit the release of inflammatory cytokines IL‐17A, IFN‐γ, IL‐1β, IL‐6, IL‐1, IL‐23, TGF‐β, granulocyte‐macrophage colony‐stimulating factor (GM‐CSF), and inflammatory chemokines CXCL1, CXCL2, CXCL9, CXCL10, CXCL11, and CCL20. It also regulates the STAT/NF‐κB pathway to inhibit the differentiation of Th1 (CD4+IFN‐γ+) cells and Th17 (CD4+IL‐17+) cells. The mechanistic study found that blocking the suppressor of cytokine signaling 3 (SOCS3) pathway in Th17 cells can reverse the inhibitory effect of baicalein on inflammatory cells, suggesting that baicalein exerts its anti‐inflammatory effects by promoting the expression of intracellular SOCS3 [169]. Xu et al. [170] found that baicalein increases the expression of peroxisome proliferator‐activated receptor β/δ (PPARβ/δ) by inhibiting the expression of intracellular 12/15‐lipoxygenase (12/15‐LO) levels, thereby inhibiting the migration of self‐reactive T cells to the CNS and the activation of microglia.

Genistein, also known as daidzein, is an isoflavone extracted from soybeans (*Glycine max* (Linn.) Merr.) and is a potent tyrosine kinase and topoisomerase inhibitor. Dias et al. [171] found that 200 mg/kg sc genistein prolongs the disease latency period in EAE mice. Further studies have shown that genistein enhances the expression of Toll‐like receptor 3 (TLR3) and TLR9, reduces the inflammatory response and demyelination in the EAE model, and exerts a preventive effect on early neurologic lesions. Another study reported that genistein at a dose of 200 mg/kg has therapeutic effects in EAE mice, downregulating the expression of IFN‐γ, TNF‐α, and IL‐10. In vivo microscopic examination found that genistein inhibits leukocyte migration and adhesion to the CNS, exerting its pharmacological effects [171].

Silymarin, a flavonoid lignan extracted from the milk thistle plant (*Silybum marianum* (L.) Gaertn.) in the Asteraceae family, has antiviral, anti‐fibrotic, anti‐tumor, and hepatoprotective effects [172]. Min et al. [173] reported that silymarin has immunomodulatory effects on the CNS of EAE mice. Intragastric administration of 10 mg/kg silymarin can reduce the severity of CNS tissue lesions in EAE mice. Further studies have shown that silymarin can dose‐dependently reduce the production of pro‐inflammatory cytokines IL‐2 and IL‐12 by Th1 cells while increasing the secretion of anti‐inflammatory cytokine IL‐4 by Th2 cells, exerting its biological effects in the treatment of EAE [173]. Navabi et al. [174] studied the regulatory effects of silymarin on Th1 cells in RRMS patients receiving IFN‐β treatment, and the results showed that this compound inhibited the levels of the transcription factor T‐bet and IFN‐γ in Th1 cells in a dose‐dependent manner, preventing the proliferation and activation of Th1 cells.

Hesperidin, also known as hesperetin‐7‐O‐rutinoside, is a flavanone found in citrus fruits and exhibits anti‐inflammatory, antioxidant, and antibacterial effects [175, 176]. Haghmorad et al. [177] found that hesperidin at doses of 50–200 mg/kg can inhibit disease progression, reduce CNS leukocyte infiltration, promote the proliferation of Treg cells, inhibit Th17 cell proliferation, and reduce IL‐17 and IL‐6 expression levels in EAE mice. Further studies revealed that hesperidin exerts its effects by downregulating the expression of the transcription factor RORγt in Th17 cells and upregulating the expression of the transcription factor Foxp3, enhancing its anti‐inflammatory effects [177]. Ciftci et al. [178] confirmed that hesperidin at a dose of 50 mg/kg mainly reduces excessive lipid peroxidation in the brain tissue of EAE mice, lowers oxidative stress levels, prevents excessive immune reactions in the CNS, decreases the levels of the cytokines TNF‐α and IL‐1β, induces the high expression of caspase‐3, and promotes the apoptosis of inflammatory cells, thus preventing and treating EAE.

Apigenin, also known as “plant estrogen,” exhibits various therapeutic effects such as anticancer, anti‐inflammatory, antioxidant, and blood lipid regulation. Ginwala et al. [179] reported that apigenin improves the severity of EAE in mice, reduces the number of relapses, prevents the accumulation of DC cells and T cells in the CNS, and reduces immune cell infiltration and demyelination in the CNS. Further studies have shown that apigenin exerts its pharmacological effects by significantly reducing the expression of integrin α4 and C‐type lectin 12A (CLEC12A) in immune cells. Alpha B‐crystallin is a small heat shock protein, and its overexpression has been associated with autoimmune diseases and neurodegenerative diseases. Verbeek et al. [180] studied the anti‐inflammatory effects of apigenin on peripheral lymphocytes in vitro and found that it significantly inhibits the expression of alpha B‐crystallin antibodies and IFN‐γ in self‐reactive T cells at concentrations ranging from 3.5 to 35 μmol/L [179–181].

Licochalcone A, a type of natural flavonoid extracted from the root and stem of *Glycyrrhiza uralensis* Fisch., has various pharmacological activities including anti‐inflammatory, antibacterial, antitumor, and antiparasitic effects. Huang et al. [182] found that licochalcone A has neuroprotective effects in a mouse model of Parkinson’s disease (PD). Further research revealed that licochalcone A exerts its effects by inhibiting the phosphorylation levels of extracellular regulated protein kinases (ERK1/2) and NF‐κB family transcription factor p65, preventing the activation of microglia and the reduction of dopaminergic neurons in the PD model. Fontes et al. [183] reported that intragastric administration of 40 mg/kg licochalcone A delays the progression of the disease and reduces clinical symptom scores in the EAE mouse model. The mechanistic study demonstrated that licochalcone A at a concentration of 40 μmol/L exerts its immunomodulatory effects by suppressing the expression of H2O2, nitric oxide (NO), IFN‐γ, TNF‐α, and IL‐17 in immune cells of EAE mice [182, 183].

Procyanidins (PCs) are natural polyphenolic compounds widely distributed in the skins, seeds, leaves, and fruits of plants—such as grape seeds, pine bark, blueberries, and purple sweet potatoes, all of which are rich in PCs. Among them, grape seed PCs (GPCs) have been extensively investigated for their pharmacological effects. Pharmacological experiments have demonstrated that GPCs ameliorate clinical symptoms in experimental autoimmune EAE mice, inhibit inflammatory cell infiltration in the CNS, and reduce demyelination. Mechanistically, GPCs suppress the expression of oxidative stress markers and inflammatory cytokines in the CNS, while downregulating the phosphorylated expression of protein kinase B (Akt), extracellular signal‐regulated kinase (ERK), and c‐Jun N‐terminal kinase (JNK). Collectively, these findings indicate that the therapeutic effects of GPCs on EAE mice are associated with the inhibition of mitogen‐activated protein kinase (MAPK) signaling pathway and phosphatidylinositol 3‐kinase (PI3K)/Akt signaling pathway, providing a theoretical basis for the clinical application of GPCs in MS [184].

Quercetin (QR), a natural flavonoid compound widely distributed in the plant kingdom, exhibits diverse pharmacological activities. As demonstrated by Chen et al. [185], QR holds potential therapeutic effects in several diseases, including MS, osteoarthritis, type 2 diabetes mellitus, and acute leukemia. Subsequently, MS was selected as a representative indication for in vivo validation. Animal studies revealed that QR significantly delays the onset of disease in a classic murine model of MS, while ameliorating inflammatory infiltration and demyelination in the CNS. Combined with network pharmacology approaches, the therapeutic mechanism of QR in MS was further validated to be associated with the expression of inflammatory cytokines (TNF‐α, IL‐6, IL‐1β, IFN‐γ, IL‐17A, and IL‐2) linked to the TNF‐α/TNFR1 signaling pathway. In summary, this study expands the clinical indications of QR and preliminarily validates its therapeutic efficacy and underlying mechanisms in MS, highlighting its potential as a natural therapeutic agent for this disease.

**Figure 6:**
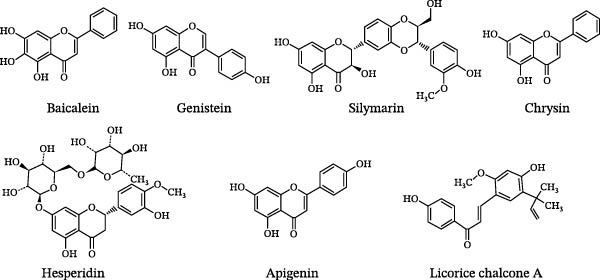
Chemical structures of flavonoids with EAE activity.

As reported by Jiang et al. [186], morroniside, a natural iridoid glycoside, possesses anti‐inflammatory and antioxidant activities, yet its therapeutic mechanisms in MS remain elusive. Notably, morroniside significantly delayed the onset of EAE, reduced clinical symptom scores, and attenuated weight loss in affected mice. Diffusion tensor imaging (DTI) demonstrated that morroniside restored the microstructural integrity of the corpus callosum and cerebellum, as evidenced by decreased values of apparent diffusion coefficient (ADC), mean diffusivity (MD), and radial diffusivity (RD), alongside increased fractional anisotropy (FA). Histopathological analyses further confirmed that morroniside diminished inflammatory infiltration and demyelination in the CNS. Mechanistically, morroniside exerted regulatory effects on peripheral immune homeostasis and neuroinflammation. Flow cytometric analysis revealed that morroniside downregulated the proportions of Th1 and Th17 cells while upregulatingTreg cells in the spleen. RT‐qPCR assays demonstrated that morroniside suppressed the expression of pro‐inflammatory cytokines (IL‐1β, IL‐6, and TNF‐α) in both brain and spinal cord tissues. Transcriptomic profiling indicated that differentially expressed genes (DEGs) following morroniside intervention were significantly enriched in biological processes such as inflammatory responses and immune regulation, as well as in signaling pathways including NF‐κB, PI3K‐AKT, and NOD‐like receptor (NLR) pathways. Western blot (WB), RT‐qPCR, and enzyme‐linked immunosorbent assay (ELISA) further validated that morroniside alleviated neuroinflammation by inhibiting the gene and protein expression of Tnfsf8 and downstream NF‐κB pathway components. Furthermore, morroniside ameliorated gut microbiota dysbiosis in EAE mice. Although it failed to fully restore Lactobacillus levels, its regulatory role in the intestinal microenvironment merits further investigation. Collectively, these findings illustrate that morroniside modulates the pathological progression of EAE through a multi‐targeted approach, encompassing the regulation of peripheral immune balance, suppression of NF‐κB‐mediated neuroinflammation, and modulation of the intestinal microenvironment—highlighting its potential as a promising natural therapeutic agent for MS. The chemical structures of flavonoids with EAE activity are shown in Figure 6.

### 4.3. Molecular Mechanisms of Triterpenoids in the Intervention of Experimental Autoimmune EAE

Triptolide, a tetracyclic diterpenoid derived from the plant *Tripterygium wilfordii* Hook. f. of the Celastraceae family, exhibits various biological activities such as anticancer and anti‐inflammatory effects. Wang et al. [187] found that intraperitoneal administration of triptolide at a dose of 100 μg/kg significantly delays brain tissue damage caused by myelin oligodendrocyte glycoprotein (MOG)‐induced EAE in mice and attenuates CNS inflammation and demyelination. Mechanistic studies have shown that triptolide exerts its therapeutic effects by inhibiting the NF‐κB pathway and upregulating the expression of the transcriptional regulatory factor Foxp3 in monocytes of the spleen [187]. Kizelsztein et al. [188] reported that 100 μg/kg triptolide increases the expression level of heat shock protein 70 (HSP70) and decreases the phosphorylation level of NF‐κB inhibitor α (IκBα) in the CNS of mice, indicating that triptolide improves CNS lesions in EAE mice by regulating HSP70 and IκBα/NF‐κB expression.

Andrographolide, a diterpenoid lactone extracted from the plant *Andrographis paniculata* (Burm. f.) Nees of the Acanthaceae family, exhibits anti‐inflammatory, antitumor, and antimicrobial effects. Relevant studies have shown that andrographolide delays the occurrence of CNS lesions and reduces the clinical symptom scores in EAE model mice at a dose of 4 mg/kg. It inhibits T cell activation and cytokine release. Further research has found that andrographolide can interfere with the NF‐κB pathway in DC cells of EAE mice, specifically inhibiting T cell activation [185, 189, 190].

Eriocalyxin B, a diterpenoid compound isolated from the plant Rabdosia eriocalyx (Dunn) Hara of the Labiatae family, exhibits anti‐inflammatory, antitumor, immunomodulatory effects, and improves acute myeloid leukemia [191]. Lu et al. [192] found that intraperitoneal administration of 10 mg/kg eriocalyxin B can alleviate CNS inflammatory responses and demyelination in EAE model mice, as well as delay the onset of the disease. Further experiments demonstrated that eriocalyxin B inhibits the JAK/STAT pathway, suppresses the phosphorylation of STAT1 and STAT4 affecting Th1/CD4+ T cell proliferation, inhibits the phosphorylation of STAT3 affecting Th17/CD4+ T cell proliferation, suppresses the NF‐κB signaling pathway and inflammatory T cell proliferation in brain and spinal cord tissues, increases intracellular reactive oxygen species (ROS) levels, improving and treating the disease in EAE mice [192]. Adenanthin, found in the plant Rabdosia adenantha (Diels) Hara of the Labiatae family, exhibits biological effects such as immune regulation and inhibition of myeloma cell proliferation. Yin et al. [193] found that adenanthin has preventive and therapeutic effects on EAE mice. Intraperitoneal administration of 20 mg/kg adenanthin can reduce the clinical scores and alleviate CNS damage in EAE model mice. Mechanistic studies have shown that adenanthin binds to the p65 subunit and IKK kinase, significantly inhibiting TNF‐α‐induced NF‐κB activation, reducing NF‐κB DNA‐binding ability, suppressing the expression of NF‐κB target genes, and affecting the proliferation of APCs in the treatment of EAE [193]. Paclitaxel, primarily sourced from the root of *Taxus chinensis* (Pilger), is a microtubule stabilizer that shows promising therapeutic effects on advanced cervical cancer and ovarian cancer. Crume et al. [194] and O’Sullivan et al. [195] reported that intraperitoneal administration of paclitaxel at a dose of 20 mg/kg for 5 consecutive days significantly reduces the incidence, delays the onset of disease, decreases neurological impairment scores, and inhibits CNS inflammatory cell infiltration in EAE model mice. Paclitaxel significantly inhibits the proliferation of CD4+ T cells, CD8+ T cells, and LN cells in EAE mice, and reduces the levels of antigen‐specific cytokines such as IL‐17 and IFN‐γ [194, 195]. Parthenolide, also known as Feverfew lactone, is a sesquiterpene lactone extracted from Chrysanthemum parthenium Pers. Carvalho et al. [196] extracted cells from the spleen and peritoneum of EAE mice and found that different concentrations (1, 5, and 20 μmol/L) of parthenolide can regulate the proliferation of Th1 and Th17 cells in vitro. The mechanism study showed that parthenolide exerts its effects by reducing the expression levels of IL‐12 p40 and IL‐6. However, the in vivo therapeutic effect of parthenolide on EAE remains to be further investigated [196]. In a study by Fiebich et al. [197], it was found that parthenolide at a concentration of 1 μg/mL can inhibit the production and release of iNOS and NO in neuronal glial cells. Further research revealed that parthenolide exerts its biological effects by inhibiting the activation of p42/p44 MAPKs [197].

Artemisinin (ART), a sesquiterpene lactone featuring a peroxo bridge structure without a heterocyclic nitrogen moiety, exhibits superior efficacy, lower toxicity, and fewer adverse reactions compared to traditional antimalarials such as chloroquine and quinine, albeit with a higher recurrence rate. Studies have shown that, in contrast to EAE model mice, mice treated with dihydroartemisinin (DHA)—a key derivative of ART—displayed reduced spinal cord damage and diminished inflammatory cell infiltration. Mechanistically, DHA enhanced the expression of cytotoxic T‐lymphocyte‐associated antigen 4 (CTLA4) and programed cell death 1 (PD1) in splenocytes. Additionally, DHA upregulated the expression of suppressor of cytokine signaling 3 (SOCS3) and the phosphorylation of signal transducer and activator of transcription 1 (STAT1). Collectively, these findings indicate that DHA ameliorates pathological symptoms in EAE mice by upregulating CTLA4 and PD1 expression on T cells via the STAT1/SOCS3 pathway, highlighting its substantial developmental potential as a therapeutic agent for MS [198]. A recent study [199] has uncovered a novel dual regulatory effect of ART and its derivatives on the Toll‐like receptor 4 (TLR4) signaling pathway. These compounds not only selectively modulate TLR4‐mediated inflammatory signaling cascades (e.g., the NF‐κB pathway) to effectively inhibit excessive microglial activation but also suppress TLR4 dimerization and endocytosis, thereby blocking downstream signal transduction and alleviating neuroinflammatory responses. This groundbreaking discovery provides a molecular basis for targeting TLR4 in the treatment of neuroimmunological diseases, including MS and AD. Tehranolide (TEH), a novel compound structurally analogous to ART, and ART itself markedly alleviated EAE symptoms. In the TEH‐treated group, significant reductions in the secretion of IL‐6 and IL‐17, as well as the gene expression of IL‐17 and IL‐1, were observed in the spinal cord; ART exerted similar but milder effects. Furthermore, both ART and TEH upregulated the expression of TGF‐β, IL‐4, and IL‐10 genes in the spinal cord, while neither treatment affected IFN‐γ expression. Both interventions significantly increased the expression of FOXP3, GATA3, myelin basic protein (MBP), and AXL. Additionally, T‐bet gene expression was reduced following TEH administration. Notably, neither compound altered the mRNA expression levels of RORγt, nestin, Gas6, Tyro3, or Mertk in the spinal cord. These results demonstrate that both TEH and ART effectively modulate genes involved in inflammation and myelination—processes critical to EAE pathogenesis. Interestingly, TEH exerted more potent therapeutic effects than ART, thereby holding promise for evaluation as a potential therapeutic intervention for MS [200].

Astragaloside IV, a tetracyclic triterpene saponin extracted from *Astragalus mongholicus* (Fisch.) Bunge or Astragalus membranaceus (Fisch.) Bunge, exhibits immunomodulatory, antiviral, and blood glucose regulation effects. He et al. [201] found that intraperitoneal administration of 20 mg/kg astragaloside IV can alleviate the degree of CNS lesion in EAE mice. Mechanistic studies have shown that astragaloside IV achieves this by lowering intracellular reactive oxygen species (ROS) levels and reducing the expression of inflammatory chemokines. It inhibits the activity of SOD1 and GSH‐Px, balancing the oxidative stress levels in the cells [201]. Liu et al. [202] and Yang et al. [203] found that astragaloside IV can inhibit the activation of microglia in the brain tissue of EAE mice and reduce the inflammatory response. Further research demonstrated that astragaloside IV enhances the activity of glucocorticoid receptors (GRs) in microglia and further regulates the phosphorylation levels of inflammation‐related cell factors such as phosphoinositide 3‐kinase (PI3K), protein kinase B (Akt), NF‐κB, and IκB mediated by glucocorticoids, thereby modulating CNS immune responses. Molecular docking experiments confirmed that astragaloside IV can bind to GRs even at relatively low molecular actions. Additionally, the addition of the glucocorticoid inhibitor RU486 can block the activation of astragaloside IV on microglia cells (BV‐2) in both in vitro and in vivo experiments [202, 203]. Astragaloside II (AS‐II), a cycloartane‐type triterpenoid glycoside with the molecular formula *C*_43_H_70_O_15_ (CAS No.: 84676‐89‐1), is isolated from the Fabaceae plants *Astragalus membranaceus* var. *mongholicus* and *Astragalus membranaceus*. Yuan et al. [204] performed in vitro oligodendrocyte precursor cell (OPC) differentiation assays and established in vivo demyelination models, including CPZ‐induced and experimental autoimmune EAE models. Drug affinity responsive target stability (DARTS) mass spectrometry, cellular thermal shift assay (CETSA), and surface plasmon resonance (SPR) assay identified and validated the p75 neurotrophin receptor (p75NTR) as the direct molecular target of AS‐II. Mechanistically, AS‐II ameliorated neurobehavioral outcomes, increased oligodendrocyte protein production, and enhanced myelin integrity by inhibiting the β‐catenin/Id2/MBP signaling pathway. Specifically, AS‐II binds to p75NTR at residues Pro253 and Ser257, stabilizes its structural conformation, and thereby promotes remyelination. Notably, in p75NTR knockout (p75NTR^−^/^−^) mice, AS‐II failed to restore myelin integrity or neurofunctional deficits, further validating its p75NTR‐dependent therapeutic mechanism [204].

**Figure 7:**
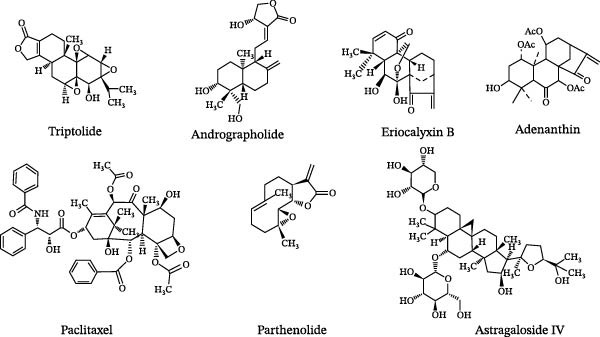
Chemical structures of triterpenoids with EAE activity.

The chemical structures of triterpenoids with EAE activity are shown in Figure 7.

### 4.4. Molecular Mechanisms of Polyphenolic Compounds in the Intervention of Experimental Autoimmune EAE

Resveratrol, a class of non‐flavonoid polyphenolic natural compounds, is found in plants such as Leguminosae, Vitaceae, and Liliaceae. It exhibits anticancer, anti‐aging, and antimicrobial effects. Singh et al. [205] found that intragastric administration of resveratrol to EAE model mice (at doses of 100 and 250 mg/kg) dose‐dependently inhibits neurologic lesions, reduces clinical scores, and downregulates the levels of cytokines (TNF‐α, INF‐γ, IL‐2, IL‐9, IL‐12, and IL‐17) and chemokines (MIP‐1α, MCP‐1) in brain and spinal cord tissues. The mechanism study revealed that resveratrol induces apoptosis of activated T cells in the CNS of EAE mice through the activation of AhR, estrogen receptor (ER), and apoptosis genes Fas/FasL [205]. Shindler et al. [206] demonstrated that resveratrol delays neuronal damage in EAE mice and the mechanism of action is related to the activation of nicotinamide adenine dinucleotide‐dependent deacetylase (SIRT1). Curcumin, derived from the plant *Curcuma longa* L. of the Zingiberaceae family, exhibits bioactivities such as anticancer, anti‐inflammatory, and antifibrotic effects. Natarajan and Bright [207] reported that intraperitoneal administration of curcumin at a dose of 50 or 100 μg/(kg·d) can inhibit immune cell activation and tissue damage in the EAE model of SJL/J mice, alleviate CNS inflammation and demyelination. Mechanistic studies demonstrated that curcumin suppresses the JAK/STAT pathway, reduces the expression of pro‐inflammatory cytokine IL‐12 in macrophages and microglia, and inhibits the differentiation of Th1 cells. Fahey et al. investigated the influence of curcumin on T cell activation and proliferation in vitro. The results showed that curcumin reduces the phosphorylation of STAT4 induced by IL‐12, and decreases the expression of IFN‐γ and IL‐12Rβ2 [208]. Natural compounds derived from saffron, such as crocins and crocetin, belong to the carotenoid family and exhibit potent antioxidant activity. Mohammadi et al. [209] employed molecular docking calculations to investigate the potential inhibitory effects of saffron‐derived natural compounds on MS. Results from biological activity assays and computational models demonstrated that gold nanoparticles (AuNPs) enhance the performance of these plant‐derived compounds, which may also participate in the inhibition of proteins implicated in MS pathogenesis [209].

Gao et al. [210] identified icaritin (ICA), a natural flavonoid compound, as an effective agent for alleviating disease progression in the experimental autoimmune EAE mouse model, including improvements in neurological deficit scores and body weight. These beneficial effects were associated with reduced demyelination in the corpus callosum and attenuated neuronal loss in the hippocampus and cerebral cortex, as confirmed by immunohistochemical analyses. Concurrently, ICA treatment inhibited microglial activation in the cerebral cortex and hippocampus, followed by downregulation of pro‐neuroinflammatory cytokines (IL‐1β, IL‐6, and TNF‐α). This anti‐inflammatory effect is likely attributed to the suppression of microglial NLRP3 inflammasome activation. Furthermore, molecular docking studies revealed a strong binding affinity between ICA and the NLRP3 inflammasome complex. Collectively, these findings indicate that ICA, as a pleiotropic agent, prevents EAE‐induced MS‐like pathology by ameliorating demyelination and neuronal loss, which is mediated through the inhibition of microglial NLRP3 inflammasome‐dependent neuroinflammation.

Han et al. [211] demonstrated that oral administration of ellagic acid (EA)—a natural polyphenol abundant in the Mediterranean diet—effectively ameliorated EAE, an animal model of MS, by regulating the microbiota‐metabolite‐immune axis. EA reshaped the composition of the gut microbiota, particularly increasing the relative abundance of SCFA‐producing genera such as *Alloprevotella*. Propionic acid (C3) was the most significantly upregulated SCFA following EA treatment, and integrative modeling revealed a strong negative correlation between *Alloprevotella* or C3 levels and EAE pathological severity. Depletion of the gut microbiota abrogated the protective effects of EA on EAE, while oral supplementation with *Alloprevotella rava* mimicked EA’s beneficial outcomes. Additionally, EA directly promoted the growth of *Alloprevotella rava* (DSM 22,548) and the in vitro production of C3. Co‐culture of cell‐free supernatant from *Alloprevotella rava* with EA inhibited Th17 cell differentiation by regulating acetylation in a cellular model. Notably, C3 itself alleviated EAE progression, potentially via inhibiting HDAC activity and upregulating acetylation, thereby reducing the secretion of pro‐inflammatory cytokines by pathogenic Th17 cells. In conclusion, this study identifies EA as a novel and promising prebiotic that improves MS and other autoimmune diseases through the microbiota‐metabolite‐immune axis [211].

Tian et al. [212] investigated luteolin (LUT), a natural flavonoid, as a dietary supplement alternative to DMF in a CPZ‐induced demyelination mouse model. LUT treatment significantly restored motor coordination and spatial memory, with efficacy comparable to that of DMF. It promoted MBP expression, mitigated oxidative damage by regulating the levels of superoxide dismutase (SOD), catalase (CAT), glutathione peroxidase (GSH‐Px), and malondialdehyde (MDA), and preserved myelin integrity. Furthermore, LUT significantly promoted Nrf2 nuclear translocation and upregulated the expression of heme oxygenase‐1 (HO‐1) and NAD(P)H quinone dehydrogenase 1 (NQO1). Molecular docking analyses revealed that LUT exhibits stronger binding affinity to the target protein Keap1 (Kelch‐like ECH‐associated protein 1) and lower potential off‐target toxicity compared to the major active metabolite of DMF. In conclusion, LUT exerts significant neuroprotective effects by activating the Nrf2 pathway, with efficacy comparable to DMF but superior safety profiles. These findings highlight LUT as a promising natural compound bridging functional foods and MS therapy, laying the foundation for the development of LUT‐rich dietary regimens as adjuvant treatments for MS.

**Figure 8:**
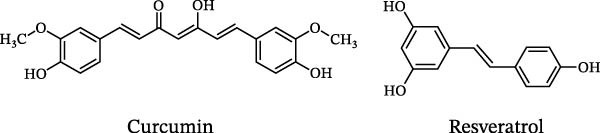
Chemical structures of polyphenolic compounds with EAE activity.

The chemical structures of polyphenolic compounds with EAE activity are shown in Figure 8.

### 4.5. Molecular Mechanisms of Naphthoquinone Compounds in the Intervention of Experimental Autoimmune EAE

Plumbagin, a natural naphthoquinone compound, has anti‐inflammatory, anticancer, and antioxidant effects. Zhang et al. [213] found that intraperitoneal administration of plumbagin at a dose of 2 mg/kg can reduce CNS inflammation and demyelination in EAE model mice. Plumbagin exerts its anti‐inflammatory effects by inhibiting the proliferation of DCs. It inhibits the differentiation, maturation, and phagocytic function of monocyte‐derived DCs, and reduces the expression levels of Th1/Th17 polarizing cytokines (IL‐12, IL‐6, IL‐1β, TGF‐β, IL‐23, IFN‐γ, and TNF‐α) in mature DCs [213]. Jia et al. [214] found that plumbagin improves the clinical scores of EAE mice by regulating the immune response of encephalitogenic T lymphocytes. Plumbagin achieves this by reducing the phosphorylation levels of JAK1/JAK2, STAT1/STAT4, transcription factors, and NF‐κB, thereby modulating Th1 cell differentiation, inhibiting the expression of inflammatory chemokines (iNOS, IFN‐γ, and IL‐6), and improving the clinical symptoms of EAE model mice [214].

Shikonin, a purplish‐red naphthoquinone natural pigment extracted from the plant *Lithospermum erythrorhizon*, has been found to improve experimental autoimmune EAE through immunomodulation, anti‐apoptosis, and antioxidant activities [215].

Rhubarb contains mainly anthraquinone compounds, including free anthraquinones such as rhein, aloe‐emodin, emodin, physcion, chrysophanol, and anthraquinone glycosides such as emodin‐8‐O‐glucoside. Emodin, a natural product derived from rhubarb and other herbal plants, exhibits various activities such as anti‐inflammatory and antioxidant effects. Current studies have found that emodin possesses neuroprotective effects in CNS diseases. It has been found that emodin can improve clinical symptoms, inhibit inflammatory cell infiltration and demyelination in the spinal cord, and suppress inflammatory cell infiltration in the brain, thereby reducing inflammation and exerting neuroprotective effects in the EAE rat model. Emodin can also inhibit the inflammatory levels in models of microglial inflammation. In the EAE rat model and microglial inflammation model, emodin can suppress NLRP3 inflammasome activation and pyroptosis. Emodin regulates NLRP3 inflammasome activation and pyroptosis through the SIRT1/PGC‐1α pathway [216]. Yang et al. [217] investigated the therapeutic potential of 2‐bromo‐1,4‐naphthalenedione (BrQ), a naphthalenedione derivative, in EAE—an animal model of MS—and elucidated its underlying mechanisms. Notably, BrQ‐treated mice exhibited alleviated EAE symptoms, including reduced clinical scores, diminished leukocyte infiltration, and attenuated demyelination in the CNS. Furthermore, the study indicated that BrQ does not directly affect the remyelination process. Through CellChat analysis based on bulk RNA‐seq data, combined with flow cytometric validation, the researchers found that BrQ significantly promoted the expansion of CD8^+^ T cells in the peripheral immune system of EAE mice and enhanced their crosstalk with other immune cell subsets. Subsequent CD8^+^ T cell depletion experiments confirmed that BrQ alleviates EAE in a CD8^+^ T cell‐dependent manner. Mechanistically, the expanded CD8^+^ T cells were found to selectively reduce antigen‐specific CD4^+^ T cells, thereby inhibiting the development of Th1 and Th17 cells in vivo and ultimately mitigating EAE. Collectively, these findings highlight the critical role of BrQ in regulating MS pathogenesis, suggesting its potential as a novel drug candidate for the treatment of MS and other autoimmune diseases [217].

### 4.6. Molecular Mechanisms of Coumarin Compounds in the Intervention of Experimental Autoimmune EAE

Daphnetin, a coumarin compound extracted from the leaves of Daphne koreanum Nakai, is a newly developed natural drug in China, primarily used clinically for the treatment of arthritis and thromboangiitis obliterans. Wang et al. [218] reported that intraperitoneal administration of daphnetin at a dose of 8 mg/kg can inhibit CNS inflammation and demyelination in EAE mice. The study confirmed that daphnetin exerts its effects by suppressing the activation, maturation, and antigen presentation function of DCs, and inhibiting the proliferation of Th1 and Th17 cells. Mechanistic studies have shown that daphnetin mainly inhibits the NF‐κB pathway, increases the expression levels of heme oxygenase‐1, an immune‐regulatory negative factor, and inhibits excessive immune responses in EAE model mice [218]. Song et al. [219] found that daphnetin has immunosuppressive effects in both in vitro and in vivo experiments. Daphnetin shortens the cell cycle, reduces the expression of inflammatory cytokines (IL‐2, IL‐4, IL‐6, and IFN‐γ), inhibits the proliferation and migration of T lymphocytes, and prevents hypersensitivity reactions in mice [219].

Osthol, also known as ostholin or ostholin methylether, is a coumarin compound extracted from the Umbelliferae plant *Cnidium monnieri* (L.) Cuss. It exhibits activities such as antioxidant and tumor inhibition. Chen et al. [220] found that osthol at a dose of 30 mg/kg can reduce CNS damage, alleviate inflammatory cell infiltration and demyelination in EAE model mice. It exerts its effects by enhancing the expression of nerve growth factor (NGF) in the CNS of EAE model mice and reducing the expression of the inflammatory cytokine IFN‐γ [220]. Gao et al. [221] studied the effect of osthol on EAE model mice using bone marrow/neural stem cell transplantation (BM‐NSCs) therapy. The results showed that osthol can inhibit the demyelinating response of BM‐NSCs in the CNS, promote the differentiation of BM‐NSCs into oligodendrocytes and neuronal cells, inhibit the formation of astrocytes, facilitate the repair of myelin/phospholipid and axonal damage in the CNS, and enhance the therapeutic effect of BM‐NSCs in the EAE model [221].

Daphnetin, an active ingredient extracted from the plant Daphne koreanum Nakai, has been found to exhibit anti‐inflammatory and neuroprotective effects in the pathogenesis of EAE. It has been observed that daphnetin treatment in EAE mice resulted in lower levels of pro‐inflammatory cytokines, including IL‐17, INF‐γ, IL‐6, IL‐12a, and IL‐23a in the brain compared to control littermates [222]. Additionally, daphnetin inhibited the production of IL‐1β, IL‐6, and TNF‐α in lipopolysaccharide‐stimulated mouse BV2 microglial cells. Mechanistically, daphnetin exerts its anti‐inflammatory and neuroprotective effects in the pathogenesis of EAE at least in part through its regulation of heme oxygenase‐1 [223]. Furthermore, daphnetin alleviates experimental autoimmune EAE by modulating DC activity, inhibiting Th1 and Th17 cells while upregulating Th2 and Tregs [223].

Cinnamoyloxy‐mammeisin (CNM), a 4‐phenylcoumarin isolated from propolis, has been found to attenuate the differentiation of Th17 cells in a concentration‐dependent manner (1, 3, and 10 μM) in vitro. This attenuation is associated with a reduction in the release of IL‐17 (35% inhibition) and interleukin‐22 (IL‐22, 51% inhibition). Exposure to CNM downregulates the expression of signature Th17 cell genes, indicating possible upstream molecular mechanisms. In terms of mechanism, CNM significantly reduces the phosphorylation of signal transducer and activator of transcription 3 (p‐STAT3) during the differentiation of Th17 cells in vitro. In vivo treatment with CNM (100 μg/kg) reduces clinical symptoms of EAE, accompanied by a decrease in demyelination, neuroinflammation, and Th17 response in the spinal cord and inguinal lymph nodes. Consistent with this, CNM also effectively attenuates the differentiation of human Th17 cells in vitro. Altogether, these findings highlight the potential of CNM as a novel molecule that regulates Th17 cells by inhibiting STAT3 signaling, thereby reducing autoimmune inflammation [224].Furthermore, extracts from *Tagetes lucida* and coumarins IC, HN, PE, DF, and SC have been found to exert neuroprotective effects by controlling motor injury and neuroinflammation. It was observed that these treatments increased the expression of IL‐4 and IL‐10 while decreasing IL‐1β and TNF‐α, thereby protecting the organs from increased vascular permeability, particularly the BBB, in mice with CPZ‐induced EAE [225].

**Figure 9:**
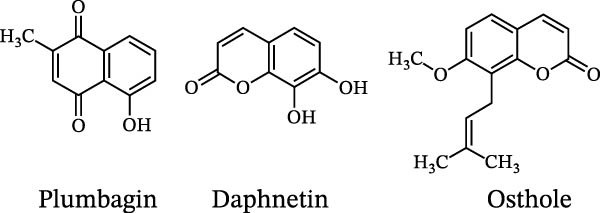
Chemical structures of compounds with EAE intervention activity.

The structures of other types of compounds with EAE intervention activity are shown in Figure 9.

### 4.7. Molecular Mechanisms of Tannin Compound Intervention in Experimental Autoimmune EAE

Urolithin, which is comprised of Urolithin A (UA) and Urolithin B (UB), is a type of natural metabolite of ellagitannins, a class of compounds called tannins, found in pomegranates and some other fruits and nuts. It has been found that oral administration of UA at a dose of 25 mg/kg/day inhibits disease progression of preclinical EAE during the preventive, induction, and effector stages. Histological evaluation revealed significantly reduced inflammatory cells, demyelination, M1 microglia, activated DC numbers, and infiltrating Th1/Th17 cells in the CNS of the UA treatment group. Treatment with 25 μM UA inhibited the activation of bone marrow‐derived DCs (BM‐DCs) in vitro, suppressed Th17 cell differentiation under T cell polarization conditions, and the DC‐CD4 T cell co‐culture system. Additionally, it was confirmed that UA inhibits the pathogenicity of Th17 cells in adoptive transfer EAE. The mechanism of action of UA is through direct targeting of the AhR and modulation of the signaling pathway. Overall, it can be concluded that UA, as a human microbial metabolite, holds therapeutic potential as a candidate drug for autoimmune diseases [226]. It has been found that EA and punicalagin, derived from pomegranate peel extract, improve neuroinflammation in mice with experimental autoimmune EAE [227].

## 5. Discussion

This review systematically synthesizes the integrative molecular and cellular mechanisms underlying MS neuroinflammation and critically evaluates the therapeutic potential of natural compounds, aiming to bridge the gap between preclinical exploration and clinical translation of natural products in MS intervention. The core argument of this work is that natural compounds, with their inherent biocompatibility, multi‐target regulatory advantages, and favorable safety profiles, represent promising candidates to complement or even reshape current MS therapeutic paradigms, particularly in addressing the unmet needs of progressive subtypes and neuroprotective therapy.

From the perspective of pathogenic mechanisms, MS is a multi‐layered heterogeneous disease driven by the dysregulation of immune networks (B/T cell subsets, cytokine cascades) and epigenetic modifications (miRNAs, lncRNAs, and DNA/RNA/histone modifications). As elaborated in Section 2, pro‐inflammatory pathways such as Th17/IL‐17, NF‐κB, and dysregulated B cell antigen presentation constitute core pathogenic nodes, while epigenetic modifications act as “regulatory switches” integrating genetic susceptibility and environmental triggers (e.g., vitamin D deficiency, smoking) to modulate disease progression. Current DMTs (Section 3) primarily target single links in these pathways (e.g., S1PR modulation, B cell depletion), leading to insufficient efficacy in progressive stages where neurodegeneration dominates. In contrast, natural compounds exhibit unique advantages in targeting multiple pathogenic nodes simultaneously: for example, curcumin and its derivatives can not only inhibit NF‐κB activation to suppress pro‐inflammatory cytokine secretion (IL‐1β, TNF‐α, and IL‐17) but also regulate Th17/Treg balance and promote microglial M2 polarization; berberine modulates miRNA networks (e.g., upregulating miR‐146a, downregulating miR‐326) to inhibit T cell overactivation and B cell autoantibody production; and resveratrol exerts neuroprotective effects by activating SIRT1 (a HDAC) to promote oligodendrocyte maturation and remyelination. This multi‐target characteristic enables natural compounds to intervene in the “immune‐neuro‐epigenetic” integrated pathogenic network of MS, which is difficult to achieve with single‐target synthetic drugs.

However, it is crucial to critically recognize the limitations of current natural compound research in MS, which hinder their clinical translation. First, preclinical evidence is heavily reliant on EAE models, which have inherent limitations in recapitulating human MS pathophysiology—including the lack of multifocal lesion dissemination, poor mimicry of progressive subtypes, and interspecies differences in immune cell function and CNS immune privilege. For example, while many natural compounds (e.g., tripterygium glycosides) have shown significant therapeutic effects in EAE models, their efficacy in human MS patients remains to be validated by high‐quality clinical data. Second, the molecular mechanisms of natural compounds are often superficially explored: most studies focus on phenotypic changes (e.g., reduced EAE scores and altered cytokine levels) without clarifying the upstream regulatory networks (e.g., key transcription factors and epigenetic crosstalk) and structure–activity relationships. For instance, the specific binding targets of flavonoids in the NF‐κB pathway and their synergistic effects with other epigenetic modifications (e.g., DNA methylation) are rarely elucidated in depth. Third, issues related to bioavailability and quality control persist: most natural compounds have poor water solubility, low bioavailability, and unstable in vivo metabolism (e.g., rapid degradation of curcumin in the liver), which limit their in vivo efficacy; meanwhile, the complex composition of natural products and batch‐to‐batch differences in extraction processes make it difficult to ensure consistent quality in preclinical and clinical studies. Fourth, clinical evidence for natural compounds in MS is extremely scarce, with only a few small‐sample, single‐center observational studies (e.g., on omega‐3 fatty acids and vitamin D supplements) and a lack of large‐sample, randomized controlled trials (RCTs) to verify their efficacy and safety.

Another key point of discussion is the complementary role of natural compounds with existing DMTs. As summarized in Section 3, current DMTs are plagued by severe side effects: for example, mitoxantrone has significant cardiac toxicity, fingolimod is contraindicated in patients with cardiovascular diseases, and monoclonal antibodies (e.g., alemtuzumab) increase the risk of infections and autoimmune diseases. Natural compounds, with their low toxicity and high biocompatibility, can potentially be used in combination with DMTs to reduce the dosage of synthetic drugs (thereby reducing side effects) while enhancing therapeutic efficacy through synergistic effects. For example, preclinical studies have shown that combining low‐dose curcumin with IFN‐β can significantly improve the therapeutic effect in EAE models compared to either agent alone, while reducing the hematological toxicity of IFN‐β. Similarly, resveratrol can enhance the remyelination effect of GA by promoting BDNF secretion. This “natural compound‐DMT combination” strategy may provide a new approach to balance efficacy and safety in MS treatment, especially for patients who cannot tolerate high‐dose DMTs.

In conclusion, natural compounds hold great promise as emerging therapeutic targets for MS due to their multi‐target regulatory effects on neuroinflammation and neuroprotection. However, their clinical translation is currently hindered by limitations in preclinical models, insufficient mechanistic exploration, poor bioavailability, and lack of high‐quality clinical evidence. To fully exploit the therapeutic potential of natural compounds, future research must prioritize addressing these limitations, with a focus on integrating preclinical data with clinical evidence, clarifying molecular mechanisms at the network level, optimizing drug delivery systems, and designing rigorous clinical trials. The discussion above lays the foundation for the subsequent “Future Perspectives” section, which will further elaborate on the specific strategies to promote the translational application of natural compounds in MS.

## 6. Future Perspectives

Based on the critical analysis of current research progress and limitations, this section focuses on three core directions required by reviewers—integrating natural products with existing DMTs, addressing challenges in clinical trial design, and exploring prospects of combination therapies—while proposing actionable strategies to advance the translational impact of natural compounds in MS treatment. These directions are interconnected and collectively aim to establish a directional framework for transforming natural compounds from preclinical candidates to clinically actionable therapeutic agents.

### 6.1. Integration of Natural Products With Existing DMTs: Synergy and Dose Optimization

The integration of natural products with existing DMTs is a promising strategy to overcome the limitations of single‐agent therapy, and its core lies in exploiting synergistic effects while reducing toxic side effects. To achieve effective integration, three key aspects need to be addressed. First, systematic exploration of synergistic mechanisms between natural compounds and DMTs is essential. This requires in‐depth studies on the molecular crosstalk between the two types of agents: for example, whether natural compounds can regulate the pharmacokinetic processes of DMTs (e.g., enhancing absorption, reducing metabolism) or synergistically target different links in the same pathogenic pathway. For instance, SIRT1 activators (e.g., resveratrol) can promote oligodendrocyte remyelination, which can complement the peripheral immune suppression effect of S1PR modulators (e.g., fingolimod), thereby achieving “peripheral immune regulation + central neuroprotection” dual effects [228]. Second, rational dose optimization based on preclinical pharmacodynamic studies is necessary. The goal is to identify the minimum effective dose combination that maximizes therapeutic efficacy while minimizing side effects. For example, in preclinical EAE models, dose‐escalation studies can be conducted to determine the optimal ratio of curcumin to IFN‐β, avoiding the increased toxicity caused by high doses of either agent alone. Third, attention must be paid to potential drug–drug interactions. Natural compounds may affect the activity of liver cytochrome P450 enzymes, thereby altering the metabolism of DMTs. For example, St. John’s wort can induce CYP3A4 activity, which may reduce the plasma concentration of teriflunomide. Therefore, preclinical pharmacokinetic studies and clinical drug–drug interaction monitoring are indispensable for the safe integration of natural products and DMTs.

In addition, the integration strategy should be personalized based on MS subtypes and patient characteristics. For RRMS patients with frequent relapses, combinations of natural compounds with strong anti‐inflammatory effects (e.g., berberine) and DMTs (e.g., ofatumumab) can be prioritized to enhance immune suppression; for SPMS/PPMS patients dominated by neurodegeneration, combinations of natural compounds with neuroprotective and remyelinating effects (e.g., resveratrol, QR) and DMTs (e.g., siponimod) may be more effective. This personalized integration requires the guidance of biomarkers, such as using cytokine profiles (e.g., IL‐17 and TNF‐α levels) or epigenetic signatures (e.g., miRNA expression patterns) to select appropriate combination regimens.

### 6.2. Challenges in Clinical Trial Design for Natural Compounds and Countermeasures

The clinical translation of natural compounds in MS is severely hindered by unique challenges in clinical trial design, which differ from those of synthetic drugs. Addressing these challenges requires innovative trial designs and rigorous quality control systems. First, the issue of sample size and patient stratification. Due to the high heterogeneity of MS subtypes and the relatively mild efficacy of natural compounds compared to DMTs, large‐sample, multi‐center RCTs are needed to detect significant therapeutic effects. Moreover, patient stratification based on clinical subtypes (RRMS/SPMS/PPMS), genetic background (e.g., HLA‐DRB1 ^∗^15 haplotype), and molecular biomarkers (e.g., cytokine levels, miRNA profiles) is essential to avoid masking efficacy differences due to population heterogeneity [229]. For example, trials targeting RRMS patients with high IL‐17 levels may be more likely to demonstrate the efficacy of natural compounds that inhibit Th17 differentiation. Second, the design of control groups. Given the lack of standardized natural compound preparations, placebo‐controlled trials are necessary to exclude the placebo effect; however, for ethical reasons, add‐on trials (natural compounds + standard DMTs vs., standard DMTs + placebo) are more feasible for patients with active MS. This design can not only evaluate the synergistic effect of natural compounds but also ensure that patients receive standard treatment. Third, the selection of clinical endpoints. Traditional endpoints such as relapse rate and EDSS score may not be sensitive enough to detect the mild to moderate efficacy of natural compounds, especially in progressive subtypes. Therefore, it is necessary to combine objective neuroimaging indicators (e.g., brain lesion volume and gray matter atrophy rate measured by MRI), neurophysiological indicators (e.g., evoked potentials), and patient‐reported outcomes (e.g., fatigue and cognitive function scales) to comprehensively evaluate therapeutic effects. Fourth, quality control of natural compound preparations. Standardization of extraction processes, purification methods, and active ingredient content is a prerequisite for clinical trials. For example, the content of curcumin in the test product should be quantified and standardized, and batch‐to‐batch consistency should be ensured through quality control standards (e.g., HPLC fingerprinting). Fifth, long‐term safety monitoring. Although natural compounds are generally considered safe, long‐term use may still have potential side effects (e.g., liver and kidney toxicity). Therefore, long‐term follow‐up (≥2 years) is needed in clinical trials to monitor adverse reactions, especially when combined with DMTs.

Another important challenge is the lack of international consensus on the clinical trial standards for natural compounds in MS. Currently, there is no unified guideline for the design, implementation, and outcome evaluation of such trials, leading to inconsistent study designs and difficult comparison of results between different studies. Therefore, it is urgent to establish international consensus guidelines, which should include requirements for natural compound standardization, patient stratification, endpoint selection, and safety monitoring, to promote the standardization and normalization of clinical trials.

### 6.3. Prospects of Combination Therapies Based on Natural Compounds

Combination therapies based on natural compounds (including combinations between natural compounds and between natural compounds and other therapeutic modalities) have broad prospects in MS treatment, as they can further enhance therapeutic efficacy by targeting multiple pathogenic links simultaneously and reduce the risk of drug resistance. First, combinations of natural compounds with complementary mechanisms. Natural compounds with different mechanisms of action can be combined to form a “multi‐target synergy” effect. For example, combining curcumin (which inhibits NF‐κB and Th17 activation) with resveratrol (which promotes SIRT1‐mediated remyelination) can simultaneously target neuroinflammation and neurodegeneration, which is particularly suitable for progressive MS. Another example is the combination of berberine (which modulates miRNA networks) and QR (which inhibits B cell autoantibody production), which can synergistically regulate the immune network. However, the design of such combinations requires in‐depth understanding of the molecular mechanisms of each compound to avoid potential antagonistic effects. Preclinical studies should first verify the synergistic effects and optimal ratio of combinations in EAE models, and then conduct clinical trials. Second, combinations of natural compounds with cell therapies or gene therapies. Cell therapies (e.g., mesenchymal stem cell transplantation) and gene therapies (e.g., CAR‐Treg therapy) are emerging therapeutic strategies for MS, and combining them with natural compounds may enhance their efficacy and reduce side effects. For example, natural compounds can be used to pre‐treat mesenchymal stem cells to enhance their immunosuppressive and neuroprotective capabilities; or natural compounds can be combined with CAR‐Treg therapy to improve the survival and functional stability of Treg cells in the MS microenvironment. Third, the development of precision combination therapies based on multi‐omics data. With the advancement of multi‐omics technologies (genomics, transcriptomics, proteomics, and epigenomics), it is possible to identify individual‐specific pathogenic signatures (e.g., subtype‐specific miRNA networks and cytokine profiles) in MS patients. Based on these signatures, personalized combination regimens of natural compounds and/or DMTs can be designed. For example, for patients with upregulated miR‐326 and high IL‐17 levels, a combination of berberine (which downregulates miR‐326) and curcumin (which inhibits IL‐17 production) can be selected [230]. This precision combination strategy can maximize therapeutic efficacy while minimizing unnecessary drug exposure and side effects.

It should be noted that the development of combination therapies also faces challenges, such as the complexity of drug interactions, increased difficulty in safety monitoring, and higher research costs. Therefore, a step‐by‐step research strategy is recommended: first, verify the safety and efficacy of two‐drug combinations in preclinical studies; then, conduct small‐sample clinical trials to evaluate feasibility; and finally, expand to multi‐drug combinations based on positive results. In addition, the use of systems biology approaches (e.g., network pharmacology, mathematical modeling) to predict and simulate the effects of combination therapies can help optimize the combination regimen and reduce research costs.

In summary, the future development of natural compounds in MS treatment lies in the effective integration with existing DMTs, the innovative design of clinical trials to overcome translational barriers, and the exploration of precision combination therapies based on multi‐omics data. Addressing these key directions will not only promote the clinical application of natural compounds but also provide new ideas for breaking the current bottleneck in MS treatment, ultimately improving the quality of life of MS patients and reducing the burden on healthcare systems.

## 7. Conclusion

In summary, this review systematically synthesizes and critically evaluates the therapeutic potential of natural compounds as emerging modulators of MS neuroinflammation, highlighting their unique advantages of multi‐targeted regulation, favorable biocompatibility, and low toxicity compared to conventional DMTs. By integrating shared pathogenic pathways (e.g., NF‐κB inhibition, Th17/Treg balance restoration, microglial polarization regulation) across diverse compound classes, we demonstrate that natural compounds can target the core immunopathological cascades of MS—from peripheral immune activation to CNS infiltration and chronic neuroinflammation—offering a promising complementary strategy to address the limitations of current DMTs, such as severe side effects, drug dependency, and inadequate efficacy in progressive MS subtypes.

We also emphasize that the translational advancement of natural compounds is constrained by inherent limitations of preclinical EAE models (e.g., poor recapitulation of progressive MS heterogeneity), incomplete characterization of compound‐specific pharmacokinetics and bioavailability, and the lack of large‐scale, subtype‐specific clinical trials. Moving forward, prioritizing three core directions will be critical to unlocking the full potential of natural compounds: (1) rational integration of natural products with existing DMTs to synergize immune regulation and neuroprotection; (2) development of novel preclinical models that better recapitulate human MS pathophysiology; and (3) design of adaptive clinical trials to validate multi‐targeted efficacy and subtype‐specific benefits.

Ultimately, this review provides a directional framework for bridging preclinical mechanistic insights with clinical translation, aiming to accelerate the development of natural compound‐based therapies as actionable, precision‐driven interventions for MS and to alleviate the global burden of this debilitating disease.

## Author Contributions

Zhiyong Long, Qianyue Yang, and Yonghe Wu are responsible for the study concept and design. Zhiyong Long, Qianyue Yang, Yonghe Wu, Lifei Wan, and Liuting Zeng are responsible for the data collection, data analysis and interpretation; Zhiyong Long and Qianyue Yang drafted the paper; Yonghe Wu, Lifei Wan, and Liuting Zeng revised the manuscript; Lingyun Sun and Liuting Zeng supervised the study.

## Funding

This research did not receive any specific grant from funding agencies in the public, commercial, or not‐for‐profit sectors.

## Disclosure

All authors participated in the analysis and interpretation of data and approved the final paper.

## Ethics Statement

Our study did not require an ethical board approval because it is a review.

## Conflicts of Interest

The authors declare no conflicts of interest.

## Data Availability

All data generated or analyzed during this study are included in this published article.
